# Dicarbonyl Induced Structural Perturbations Make Histone H1 Highly Immunogenic and Generate an Auto-Immune Response in Cancer

**DOI:** 10.1371/journal.pone.0136197

**Published:** 2015-08-28

**Authors:** Abdul Rouf Mir, Moin Uddin, Farzana Khan, Khursheed Alam, Asif Ali

**Affiliations:** Department of Biochemistry, Jawaharlal Nehru Medical College, Faculty of Medicine, Aligarh Muslim University, Aligarh, Uttar Pradesh, India; Peking University Health Science Center, CHINA

## Abstract

Increased oxidative stress under hyperglycemic conditions, through the interaction of AGEs with RAGE receptors and via activation of interleukin mediated transcription signalling, has been reported in cancer. Proteins modifications are being explored for their roles in the development and progression of cancer and autoantibody response against them is gaining interest as a probe for early detection of the disease. This study has analysed the changes in histone H1 upon modification by methylglyoxal (MG) and its implications in auto-immunopathogenesis of cancer. Modified histone showed modifications in the aromatic residues, changed tyrosine microenvironment, intermolecular cross linking and generation of AGEs. It showed masking of hydrophobic patches and a hypsochromic shift in the in ANS specific fluorescence. MG aggressively oxidized histone H1 leading to the accumulation of reactive carbonyls. Far UV CD measurements showed di-carbonyl induced enhancement of the alpha structure and the induction of beta sheet conformation; and thermal denaturation (Tm) studies confirmed the thermal stability of the modified histone. FTIR analysis showed amide I band shift, generation of a carboxyethyl group and N-Cα vibrations in the modified histone. LCMS analysis confirmed the formation of Nε-(carboxyethyl)lysine and electron microscopic studies revealed the amorphous aggregate formation. The modified histone showed altered cooperative binding with DNA. Modified H1 induced high titre antibodies in rabbits and the IgG isolated form sera of rabbits immunized with modified H1 exhibited specific binding with its immunogen in Western Blot analysis. IgG isolated from the sera of patients with lung cancer, prostate cancer, breast cancer and cancer of head and neck region showed better recognition for neo-epitopes on the modified histone, reflecting the presence of circulating autoantibodies in cancer. Since reports suggest a link between AGE-RAGE axis and carcinogenesis, glycoxidation of histone H1 and its immunogenicity paves ways for understanding role of glycoxidatively damaged nuclear proteins in cancer.

## Introduction

Cancer is one of the deadliest diseases responsible for a large number of deaths across the globe and its early detection occupies the centre stage in reducing its overall public impact [1–2]. In this regard, identification and evaluation of autoantibodies to modified proteins in cancer patients holds prominence in biomarker development for early detection of the disease. Various post-translational protein modifications (PTMs) occurring during the development of cancers are assumed to be significant for their diagnostic relevance [3–4].

Details of PTMs, like the formation of advanced glycation end products (AGEs) with role in the development and progression of cancers are also emerging [5]. It has been reported that cancerscreate a favourable the environment for the production of AGEs because of their higher uptake of glucose to fulfil their energy needs[6–8]. The glycation products formed have the potential to bind the macrophages through the macrophage scavenger receptor and, to RAGEs and thus contribute in cancer development through their pro-inflammatory capabilities and by exploiting the requirement for the activation of interleukin 6 (IL-6)–mediated mitochondrial signal transducers and activators of transcription 3 (STAT3) [9–11]. Epidemiological evidences on the molecular heterogeneity of cancers reveal genotoxic effects of acute carbonyl stress, making diabetes patients prone to various forms of cancer [12]. AGEs induced genotoxicity in tubule cells with possible implications in enhanced cancer development in advanced kidney diseases also points towards the same correlation [13].

The detection of autoantibodies generated against aberrantly processed proteins in cancer that are immunogenic and stimulate cellular and humoral immune response have led to a series of researches aimed at the detection of cancer autoantigens on the pattern of rheumatoid arthritis, wherein anti IgG antibodies have been reported as a diagnostic biomarker [14]. Among the proteins, post-translational modifications of histones, in particular have an important role in gene expression and consequently in cancer development and progression, and their modifications are also being explored as potential biomarkers of disease progression and prognosis [15–16].

Furthermore, among the glycating agents methylglyoxal (MG), a dicarbonyl compound generated by various metabolic pathways has been identified as a major precursor in modification of various proteins, with 50,000 times morereactivity than that of glucose, with both intracellular and extracellular proteins, at physiological concentration [17] as well as at higher concentrations [18] and has been associated with a role in a plethora of diseases [9, 19]. Methylglyoxal mediated perturbations may induce structural and functional changes in the nuclear protein histone H1 with possible implications in the immuno-biology of various types of cancers.

In this study, histone H1 was incubated with increasing concentrations of methylglyoxal to generate AGEs. MG induced structural changes in the histone H1 were analysed by UV, fluorescence and CD spectroscopy, poly acrylamide gel electrophoresis, Fourier transform infrared spectroscopic analysis (FTIR), carbonyl content determination, surface Hydrophobicity (H_**0**_) estimation, Liquid chromatography mass spectroscopy (m/z analysis), scanning electron microscopy, and by studying the changed interactions in binding with DNA. The possible immunogenicity of native and the modified histones was ascertained in rabbits. Circulating autoantibodies present in patients withvarious types of cancers were assessed for their binding to native and MG modified histone H1 through direct binding and competitive inhibition studies in ELISA and by the gel retardation assay.

## Materials and Methods

Histone H1, 2,4-dinitrophenyl hydrazine (DNPH), 1-anilinonaphthalene-8-sulfonic acid (ANS), sodium dodecyl sulfate (SDS), methylglyoxal, aminoguanidine hydrochloride, diethylene triamine penta-acetic acid (DTPA), sodium azide, ethidium bromide, protein A-agarose (2.5ml pre-packed column), agarose, sodium azide, Tween-20, dialysis tubings, anti-human and anti-rabbit IgG, alkaline phosphatase conjugate, para-nitrophenyl phosphate, Freund’s complete and incomplete adjuvants were purchased from Sigma Chemical Company, St. Louis, MO, USA. Acrylamide, bisacrylamide, ammonium persulfate (APS) and N,N,N′,N′- tetramethylethylenediamine (TEMED) were from Bio-Rad Laboratories, U.S.A. Sodium hydroxide, Ethylenediaminetetraacetic acid (disodium salt), methanol, glacial acetic acid, iso-propanol, sodium chloride, sodium carbonate, sodium nitrite, silver nitrate, xylene, sodium hydroxide, formaldehyde, sodium bicarbonate, ethanol, ammonium sulphate and ammonium persulphate were obtained from Qualigens, India. Polystyrene microtitre flat bottom ELISA plates and modules were purchased from NUNC, Denmark. All other chemicals/reagents were of the highest analytical grade available.

### Determination of the Protein Concentration of histone H1

Molar extinction coefficient of calf thymus histone H1 (1280 M^-1^cm^-1^) was used to determine the concentration of the protein by measuring the absorbance of protein solutions at 280 nm. Calf thymus histone H1 molecular weight used for calculations was 21500 Dalton.

### Modification of H1 histone by methylglyoxal

The procedure given by Roberts *et al*. [20] was followed. Histone H1 modification was carried out in phosphate buffer saline (10 mM sodium phosphate buffer, pH 7.4 containing 150 mM NaCl). Histone H1 (42 μM) samples were modified by incubation with varying concentrations of methylglyoxal (2.5, 5, 7.5 and 10 mM) for 24 hours at 37°C. All the samples were extensively dialysed after incubation.

### Absorbance spectroscopy

The absorption profile of native and methylglyoxal-modified H1 histones was recorded on Shimadzu Spectrophotometer (UV 1700 model) in the wavelength range of 250–500 nm using quartz cuvette of 1 cm path length at constant temperature.

### Fluorescence studies

Fluorescence spectra were recorded on Shimadzu (RF-5301-PC) spectrofluorophotometer at 25±0.1°C in a 1 cm path length cell at 3 nm slit width.

We measured intrinsic fluorescence by exciting the protein samples at 275 nm and recording the emission spectra in 300–400 nm range.

Loss in the fluorescence intensity (F.I.) was calculated using the following equation:

$$\text{Percent loss in F}.\text{I}.=\frac{\text{F}.\text{I}.\;\text{of native sample}-\text{F}.\text{I}.\;\text{of glycated sample}}{\text{F}.\text{I}.\;\text{of native sample}}\;\text{X}\;100$$

### Sodium dodecyl sulphate-polyacrylamide gel electrophoresis

Methylglyoxal mediated modifications in histone H1 were analysed by using non denaturant non reducing vertical sodium dodecyl sulphate-polyacrylamide gel electrophoresis (PAGE) as described by *Laemilli*, with slight modifications [21]. 25 μg of native and modified histone samples were mixed with one-fourth volume of sample buffer (50% glycerol, and 0.002% bromophenol blue in 1 M tris HCl, pH 6.8) and applied into the wells of 10% SDS polyacrylamide gel. The gel was run at 80 volts for 4 h at room temperature and then stained with silver nitrate and photographed by Molecular Imager Gel Doc XR system.

Further studies were carried out on histone H1 modified by 7.5 mM methylglyoxal with native histone H1 serving as control.

### AGE Assay

AGE-specific fluorescence was measured at 440 nm after excitation at 370 nm and the increase in fluorescence intensity (F.I.) was computed by the following equation:

$$\text{Percent increase in F}.\text{I}.=\frac{\text{F}.\text{I}.\;\text{of glycated protein}-\text{F}.\text{I}.\;\text{of native protein}}{\text{F}.\text{I}.\;\text{of glycated protein}}\;\text{X}\;100$$

### Determination of Surface Hydrophobicity (H_0_)

The surface hydrophobicity was determined with the fluorescent probe 1-anilinonapthalene- 8-sulfonic acid (ANS) by fluorescence spectroscopy as per an established protocol [22]. The molar ratio between H1 histone and ANS was taken as 1:10 and emission spectra were recorded between 400–600 nm. Percentage change in fluorescence intensity (FI) was calculated by the following equation:

$$\text{Percent decrease in F}.\text{I}.=\frac{\text{F}.\text{I}.\;\text{of native protein}-\text{F}.\text{I}.\;\text{of glycated protein}}{\text{F}.\text{I}.\;\text{of native protein}}\;\text{X}\;100$$

### Determination of reactive carbonyl content

The critical evaluation of protein carbonyl content serves as biomarkers of protein oxidative damage. We analysed the carbonyl content of native and methylglyoxal modified histone H1 (MG-H1) as per the published protocol with slight modifications [23]. Native and methylglyoxal (7.5 mM) modified histone H1 samples were added with equal amounts of 10 mM DNPH in 2.5 M HCl. Samples were vortexed at regular intervals and incubated in the dark for 15 minutes at room temperature. These were incubated with 10% TCA for 15 minutes at –20°C and centrifuged at 4°C for 15 minutes at 9000 g. The protein pellets were washed three times with ice cold ethanol/ethyl acetate (1:1) after discarding the supernatant and centrifuged for 2 min at 9000 g between all washes. After the last wash, the pellet was suspended in 1 ml of 6 M guanidine-HCl and mixed properly. Protein samples were then fully dissolved by leaving at 37°C for 15–30 min. Once the protein pellets were fully dissolved, the concentration of DNPH was determined by absorbance measurement at 360 nm against guanidinium chloride (as blank) using the molar extinction coefficient of 22000 M^-1^cm^-1^. The absorbance of the samples was taken at 276 nm and protein carbonyl content was expressed as nmole mg^-1^ of protein.

### Circular Dichroism Determination

Circular Dichroism evaluates the changes in protein secondary structure, folding and their binding properties. We carried out the Far-UV CD spectral analysis of native histone H1 and its MG modified counterpart using J-815 JASCO spectropolarimeter. The instrument was attached to a microcomputer with a thermostatically controlled cell holder attached to Neslab's RTE 110 water bath with a temperature accuracy of ±0.1°C. The scans were taken at wavelength intervals of 1 nm at 25°C. All scans were recorded at wavelength intervals of 1 nm. Spectra were collected at 50 nm min^-1^ scan speeds, 0.1 nm data pitch and a response time of 2s. The spectra were recorded in far UV region between wavelengths 190 to 250 nm and the protein concentration was 0.2 mg/ml. The results were obtained in molar residual ellipticity (θ) (deg/cm^2^/dmol) at a wavelength λ, based on the equation given below [24].

$$MRE=\frac{\theta_{obs}(\text{m}\;\text{deg})}{10\times \text{n}\times \text{Cp}\times 1}$$

Where θ_obs_ is the observed ellipticity in degrees, *C*_p_ is the molar fraction and *l* is the length of the light path in centimeter. The α-helical content of different histone H1 samples were calculated from θ values at 222 nm (MRE _222_) using the following equation.

$$\%\;\alpha Helix\;=\frac{{MRE}_{222}-2340}{30300}$$

### Thermal Denaturation Studies (Tm) by Far-UV CD

The thermo-stability of the secondary structure of histone H1 and its MG modified form was analysed by Far-UV CD spectroscopy at 222 nm from 20°C to 90°C using a temperature slope of 1°C/min.

### Fourier Transform Infrared Spectroscopic Analysis—Attenuated Total Reflectance (FTIR-ATR)

Fourier transform infrared (FTIR) spectroscopy was carried out to analyse the secondary structure of histone H1 and MG modified histone using Perkin Elmer FT-IR spectrophotometer. Infrared spectral readings were recorded between 1000 cm^-1^ to 4000 cm^-1^. FTIR studies were carried out at 10 mg/ml of protein concentration.

### Liquid chromatography mass spectroscopy (m/z analysis)

The HPLC system was connected to a time-of-flight mass spectrometer (LCMS-IT-TOF, Shimadzu) equipped with an electrospray interface operated in a positive ion mode with voltage set at 4.5 kV. The temperature of the turbo ion gas was 250°C. Full scan MS analysis was carried out in the range of 0–240 m/z ratio. The MS results obtained for histone proteins were compared to that of CEL standard [25].

### Scanning Electron Microscopy (SEM) studies

The micro-architectural details of native and MG modified histones were observed using scanning electron. Air dried samples were adsorbed onto cellulose ultrafiltration membrane. The samples were coated with gold and mounted on a carbon tape coated stainless steel grids operating on an accelerating voltage of 15 kV and in low vacuum condition. The images were taken using a JSM-6510LV (JEOL JAPAN) scanning electron microscope running [26].

### Changes in interaction with DNA: circular dichroism (CD) analysis

Interaction of native and MG modified histone H1 with DNA was analysed by CD spectroscopy. 50 μg/ml of DNA was incubated for 1 hour with 0.2 mg/ml of native H1 and MG modified H1 respectively in 0.05 M tris buffer, pH 7.4. The samples were subjected to centrifugation at 14000 rpm for 8 minutes and CD spectra were recorded for the supernatant. Native DNA of same concentration was taken as control. To avoid any interference from protein, all the measurements were taken at longer wavelengths between 220 cm^-1^ to 330 cm^-1^.

### Immunological studies and Western Blotting

Randomly bred female New Zealand white rabbits weighing 1–1.5 Kg were selected for immunizationas per the established protocol of our lab [27]. Serum immunoglobulin G (IgG)from the experimental animals was isolated by affinity chromatography on protein A-agarose column [28] and Western blotting was performed as per the protocol given in the technical booklet for ECL Plus Western Blotting system (Amersham, UK) to establish the specificity of antibodies generated against native and modified histones H1 in the rabbits [29].

### Collection of blood samples for clinical Studies

In order to probe the presence of auto-antibodies against native and MG modified histone H1, sera were obtained from cancer patients (n = 83) of different age groups attending the J.N. Medical College Hospital, Aligarh, India, after the informed consent. The samples were obtained after careful clinical examination of the patients with proven histopathological diagnosis. The population included lung cancer patients (n = 27), prostate (n = 19), breast (n = 22) and patients having cancers of head and neck region (n = 15).There were 49 females with a mean age of 38.5±22.9 years and 34 males of 36±18.3 year mean age. The age of all the patients fell between 15 to 63 years. Samples from healthy individuals (n = 40; 20 males & 20 females with a mean age of 36±18.6 years) served as control. The serum samples were heated at 56°C for 30 min to inactivate complement proteins and stored at -20°C with 0.2% sodium azide. Serum antibody binding was evaluated by ELISA.

### Ethics Statement

This study was duly approved by the Institutional Ethical Committee (1617/FM/22-01-2013) and Animal Ethical Committee (8289/OAH/21-02-2013), duly registered under Committee for the Purpose of Control and Supervision of Experiments on Animals (CPCSEA) India (Registration no. 401/RO/C/2001/CPCSEA), atthe JN Medical College, Faculty of Medicine, AMU.

Blood samples were collected from the patients and healthy subjects after informed verbal consent. The mode of consent was duly approved by the ethical committee. A proper record of all the patients and healthy individuals has been maintained.

### Isolation of IgG by affinity chromatography

Serum immunoglobulin G (IgG)was isolated by affinity chromatography on protein A-agarose column [28]. Serum (0.5 ml) diluted with equal volume of PBS (pH 7.4) was applied on top of the column pre-equilibrated with the same buffer. The wash through was recycled 2–3 times and unbound material was removed by extensive washing with PBS. The bound IgG was eluted with 0.58% acetic acid in 0.85% sodium chloride and collected in a tube containing 1.0 ml of 1.0 M Tris–HCl (pH 8.5). 3 ml fractions were collected and read at 280 nm. The IgG concentration was determined considering 1.4 OD280 = 1.0 mg IgG ⁄ ml. The isolated IgG was dialysed against PBS and stored at -20°C with 0.1% sodium azide.

### Direct Binding ELISA

ELISA was carried out on flat bottom polystryrene plates as described earlier [27]. Polystryrene polysorp microtitre wells were respectively coated with 100 μl of native or methylglyoxal modified proteins (10 μg/ml). The absorbance (A) of each well was monitored at 410 nm on an automatic microplate reader. Each sample was run in duplicate and the results were expressed as mean of two readings.

### Competition ELISA

The antigenic specificity of the antibodies was determined by competition ELISA [27].Varying amounts of inhibitors (0–20 μg/ml) were mixed with a constant amount of affinity purified IgG and the mixture was incubated at room temperature for 2 hr and overnight at 4°C. The immune complex thus formed was coated in the wells in place of the IgG. The remaining steps were same as in direct binding ELISA. Percent inhibition was calculated using the formula:

$$\text{Percent inhibition}=1-\frac{A_{inhibited}}{A_{uninhibited}}\times 100$$

### Band shift assay

For the visual detection of antigen antibody binding and immune complex formation, gel retardation assay was performed [30]. Immune complexes were prepared by incubating constant amount of native and MG-modified histones with varying amounts of affinity purified immune IgG from sera of cancer patients in TBS for 2 h at 37°C and overnight at 4°C. One-fourth of sample dye was added to the mixture and electrophoresed on 10% SDS-PAGE for 4 hr at 80 V. The gels were visualized using silver nitrate staining [21].

### Statistical analysis of Results

All measurements were done in duplicates. Results are expressed as mean ± S.D. A two tailed *p* value lower than 0.05 was taken to be significant.

## Results

### Absorbance spectroscopy

**Fig 1:**
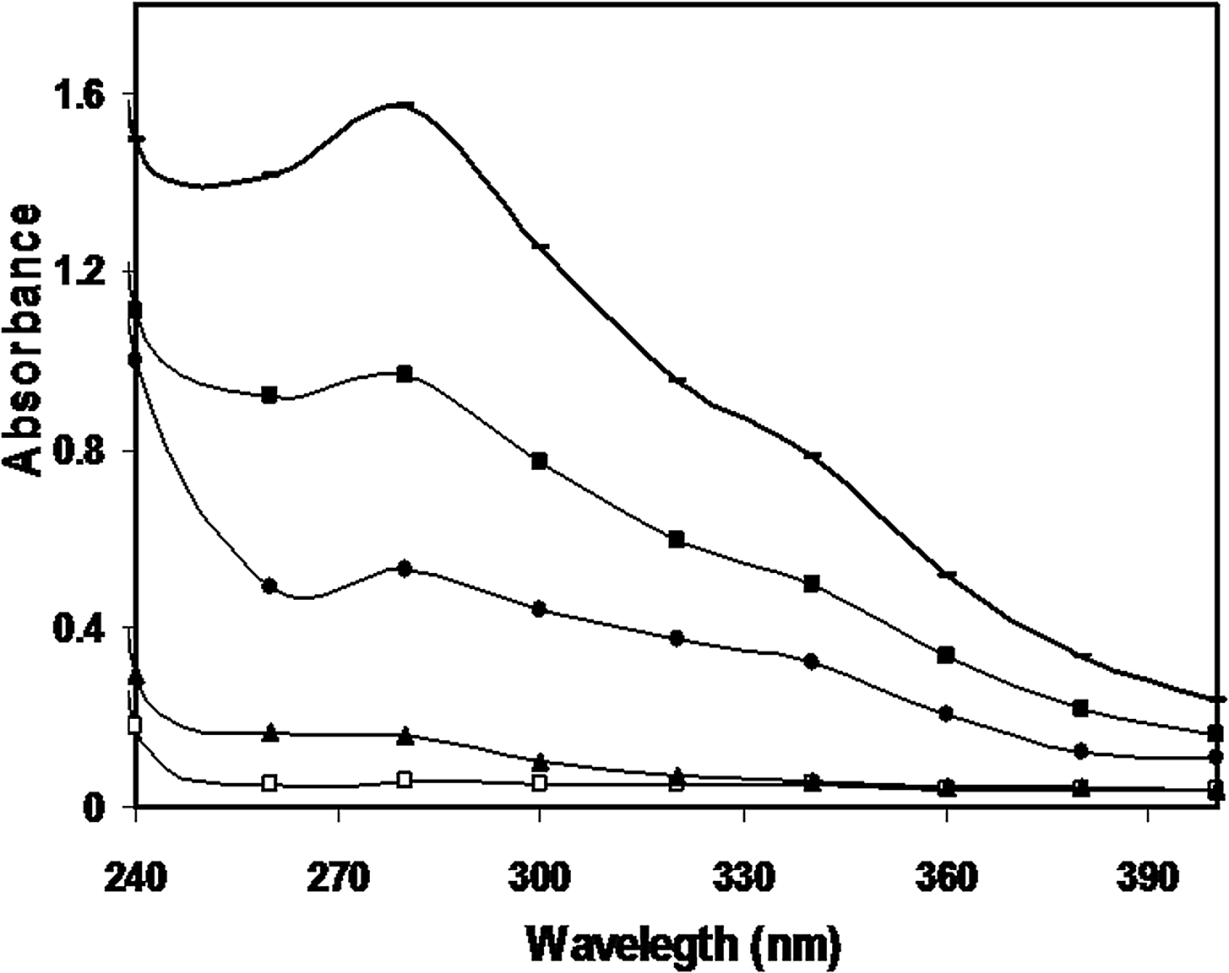
UV profile of native histone H1 (-□-), and histone H1 modified with 2.5 mM (-▲-), 5 mM (-●-), 7.5 mM (-■-) and 10 mM (-_-), methylglyoxal.

UV absorption spectrum of native histone H1 showed a characteristic peak for proteins at 277 nm. The histone H1 modified by 2.5, 5, 7.5 and 10 mM of methylglyoxal exhibited 67.87%, 90.16%, 94.54% and 96.64% hyperchromicities in the spectral peaks respectively. A hump like peak became prominent at 340 nm in the modified proteins at the higher concentrations of MG. The results are shown in Fig 1.

### Intrinsic Fluorescence

**Fig 2:**
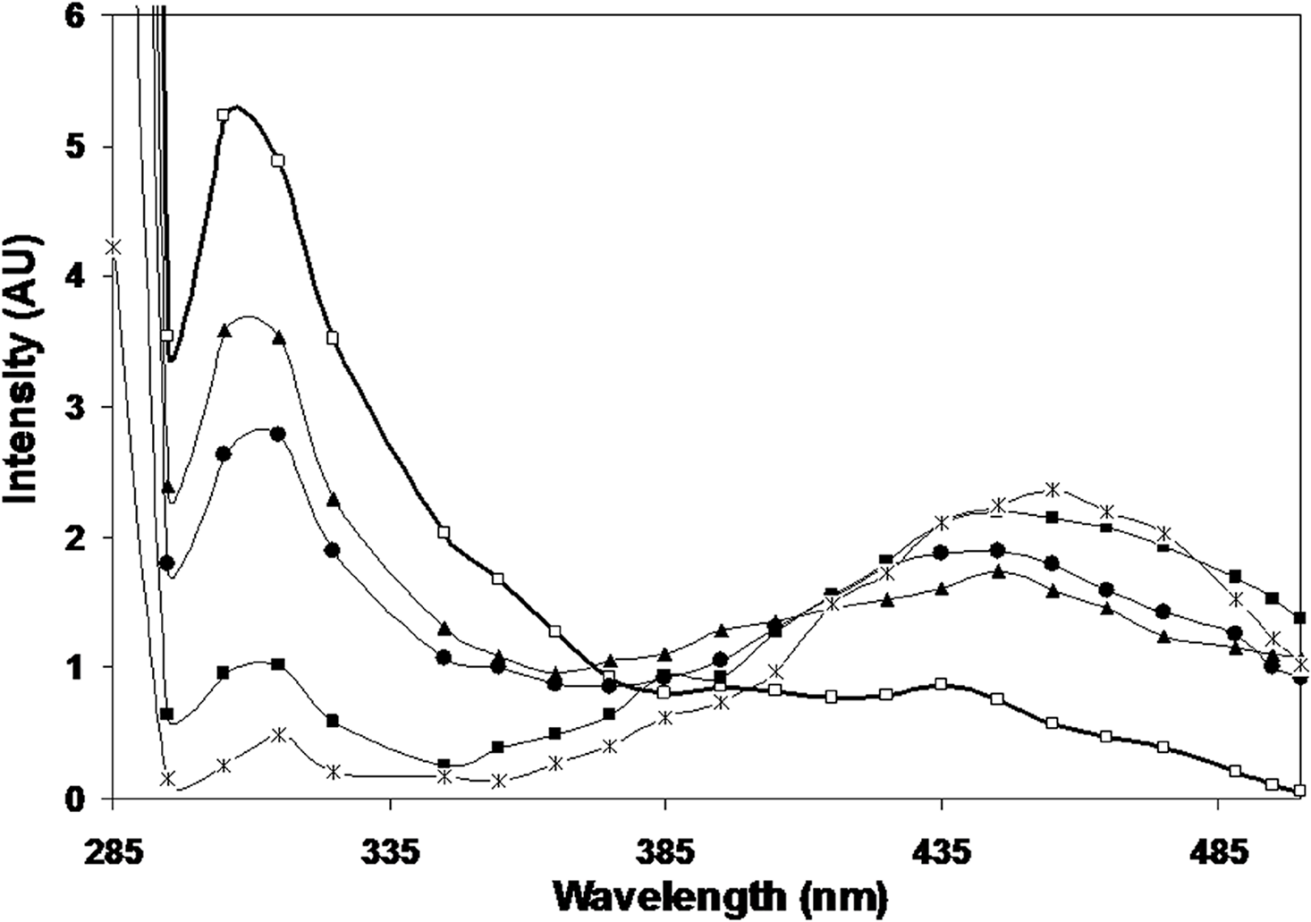
Fluorescence profile of native histone H1(-□-), and histone H1 modified with 2.5 mM (-▲-), 5 mM (-●-), 7.5 mM (-■)-) and 10 mM (-*-), methylglyoxal.

The fluorescence maxima for native histone H1 was obtained at 305 nm—a characteristic feature of tyrosine emission. The incubation of histone H1 with increasing concentrations of methylglyoxal led to a substantial decrease in fluorescence intensity. The 2.5, 5, 7.5 and 10 mM methylglyoxal modified H1 histone exhibited a decrease of 31.31%, 49.68%, 81.78% and 95.31% in intrinsic fluorescence intensity when compared to native H1 histone. The increasing concentration of methylglyoxal in the protein samples led to a red shift of 3, 6, 8 and 10 nm in emission wavelength. Furthermore, additional hump like peaks were observed in the modified protein at 440 nm. The results are shown in Fig 2.

### Electrophoretic Analysis

**Fig 3:**
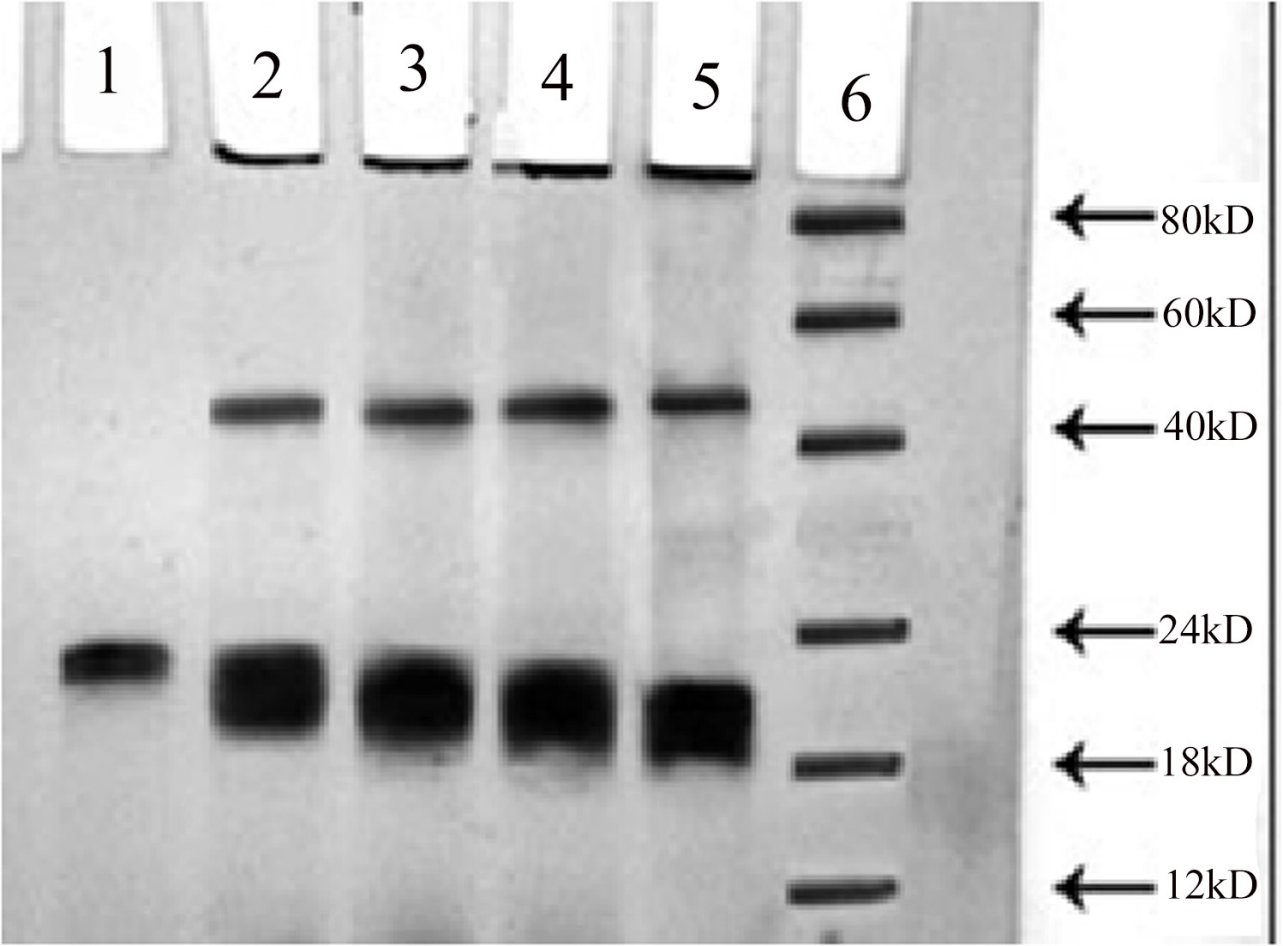
PAGE: PAGE analysis of native and MG modified histone H1: 25 μg each of native histone H1 and 2.5, 5, 7.5 and 10 mM methylglyoxal modified counterparts were loaded into the wells of 10% polyacrylamide gel under non denaturing conditions (lane 1–5), lane 6 shows the molecular weight marker.

PAGE results showed an increased electrophoretic mobility in the modified protein samples in comparison to the native histone. The modified proteins presented an increased band stretching as against the native histone. An extra band also appeared in the modified proteins at a molecular weight higher than that of native histone H1. The results are presented in Fig 3.

### AGE assay

**Fig 4:**
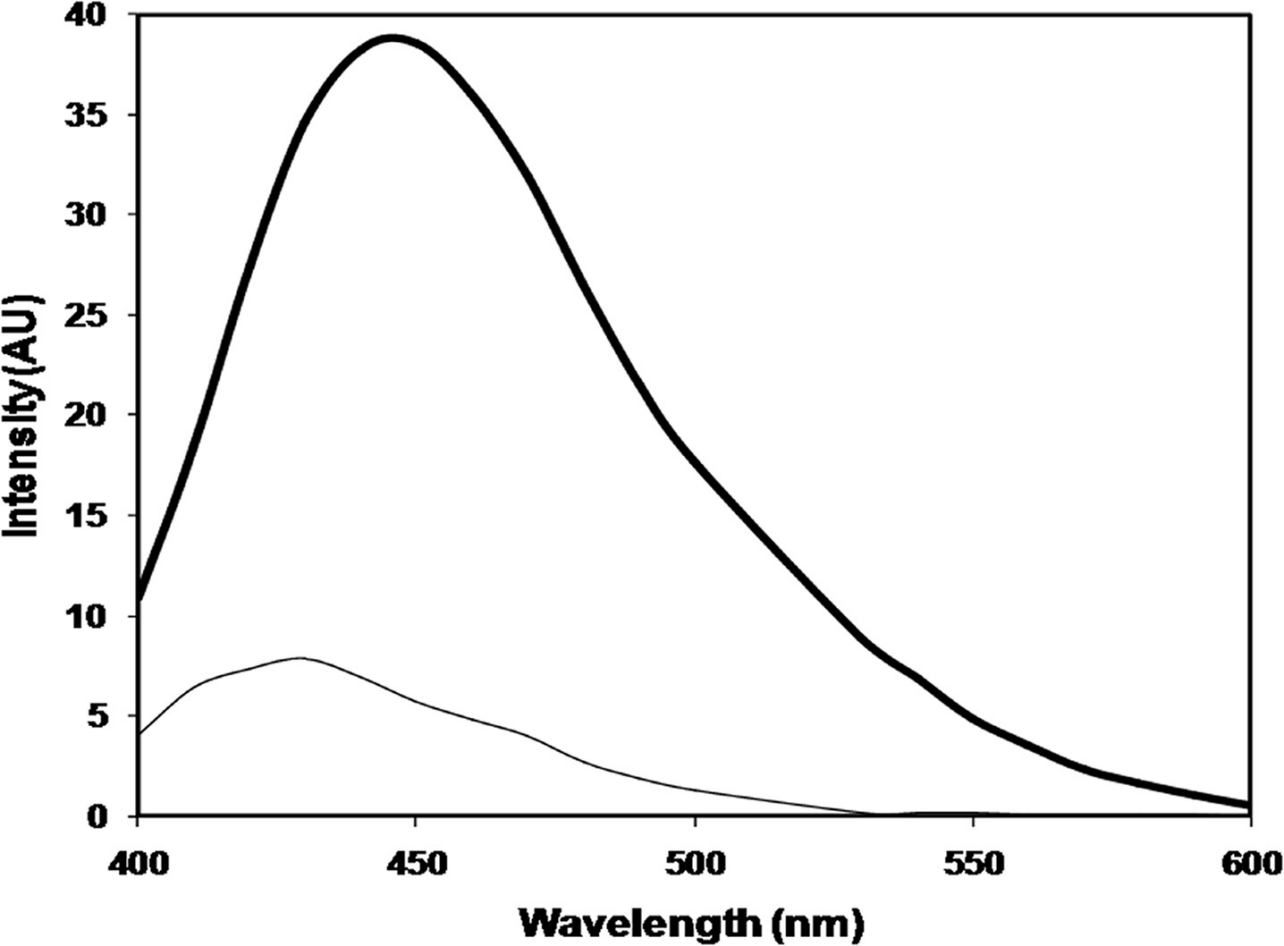
AGE fluorescence profile of native histone H1(thin line), and histone H1 modified with 7.5 mM methylglyoxal (thick line).

Under identical conditions, native histone H1 gave no appreciable AGE fluorescence. The observed fluorescence intensities for native and modified histone H1 were 7.886 at 430 nm and 39.21 at 446 nm respectively. MG-H1 exhibited 79.88% increase in AGE fluorescence intensity. The results are shown in Fig 4.

### Surface hydrophobicity (H_0_)

**Fig 5:**
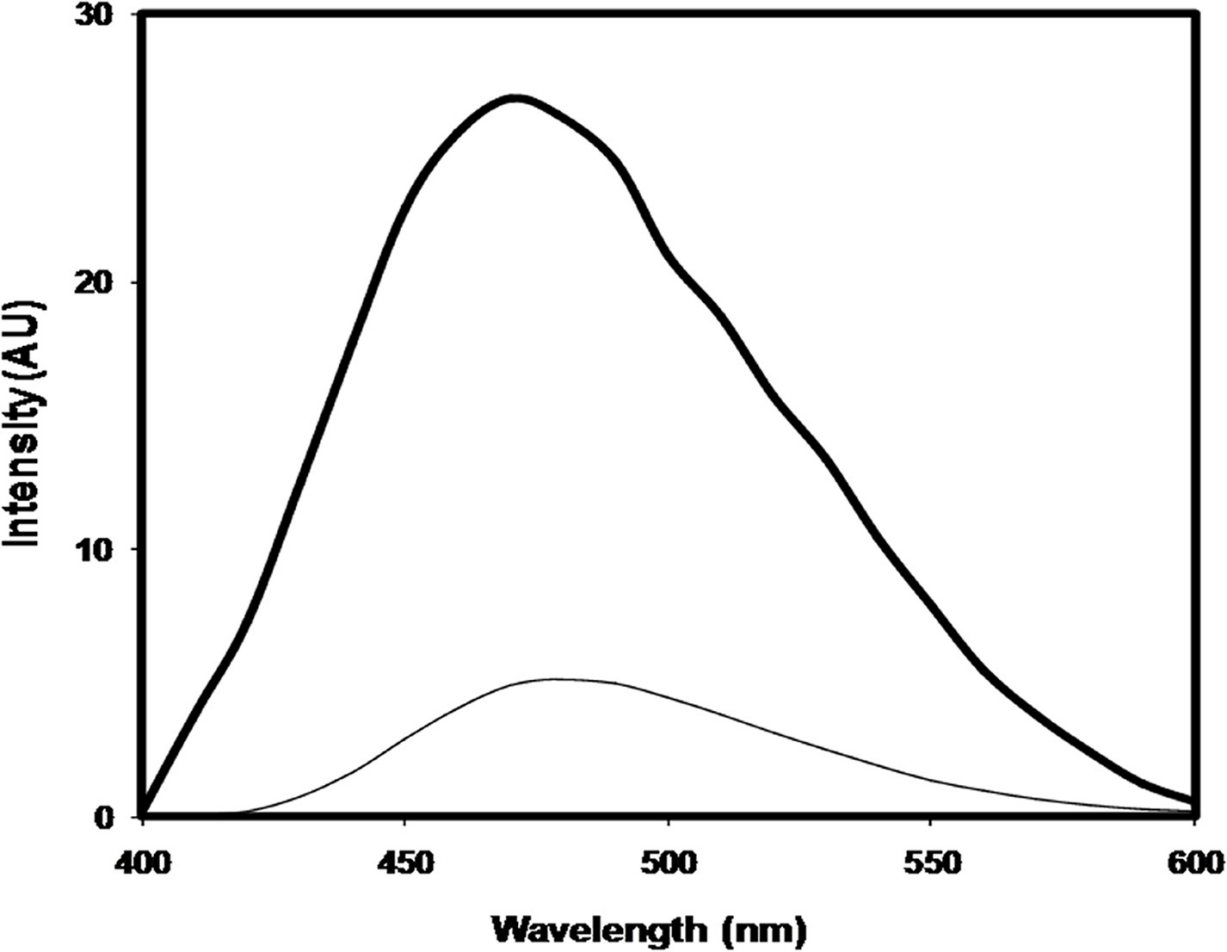
ANSfluorescence profile of native histone H1(thick line), and histone H1 modified with 7.5 mM methylglyoxal (thin line).

A significant decrease in the ANS fluorescence intensity in MG modified H1 in comparison to native histone was observed. Native and MG modified H1 showed fluorescence intensities of 26.12 at 279 nm and 5.21 at 276 nm which corresponds to a decrease in ANS fluorescence intensity by 79.97% in case of the modified histone. A blue shift of 3 nm was also seen in the case of modified protein. The results are shown in Fig 5.

### Reactive carbonyl content

**Fig 6:**
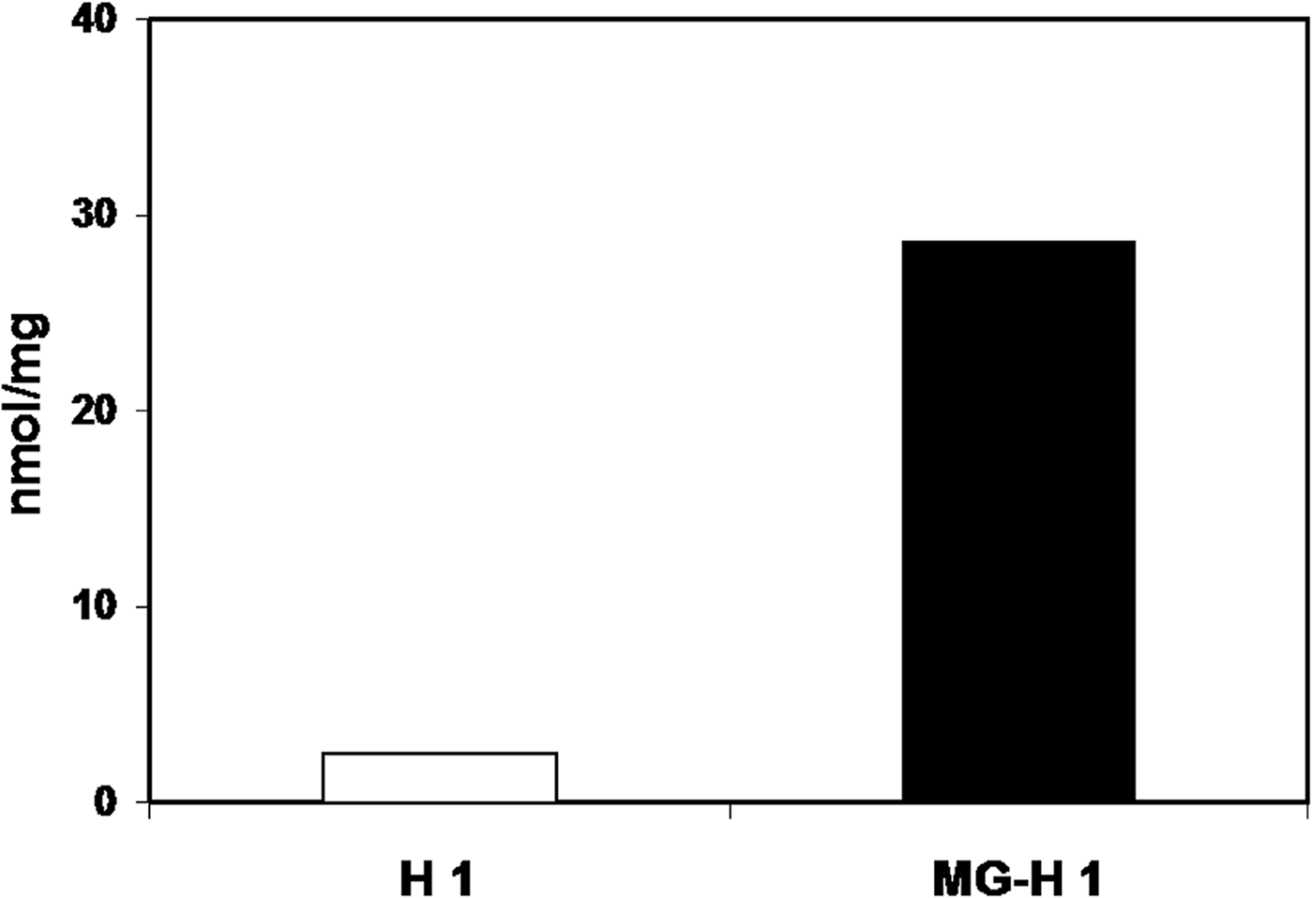
Protein-bound carbonyl concentration in native (H1) and MG modified histone (MG-H1).

The spectrophotometric quantification of reactive protein bound carbonyls after reaction with 2,4-dinitrophenylhydrazine (DNPH) showed absorbance values of 0.055 and 0.624 for native and modified histone H1 respectively at 370 nm. The carbonyl content was 2.47 nmole ⁄mg of protein in native histone H1 and 28.36 nmole ⁄mg of protein in the case of methylglyoxal modified histone H1 depicting an 11.48 times increase in reactive carbonyls. The results are shown in Fig 6.

### Circular dichroism spectra

**Fig 7:**
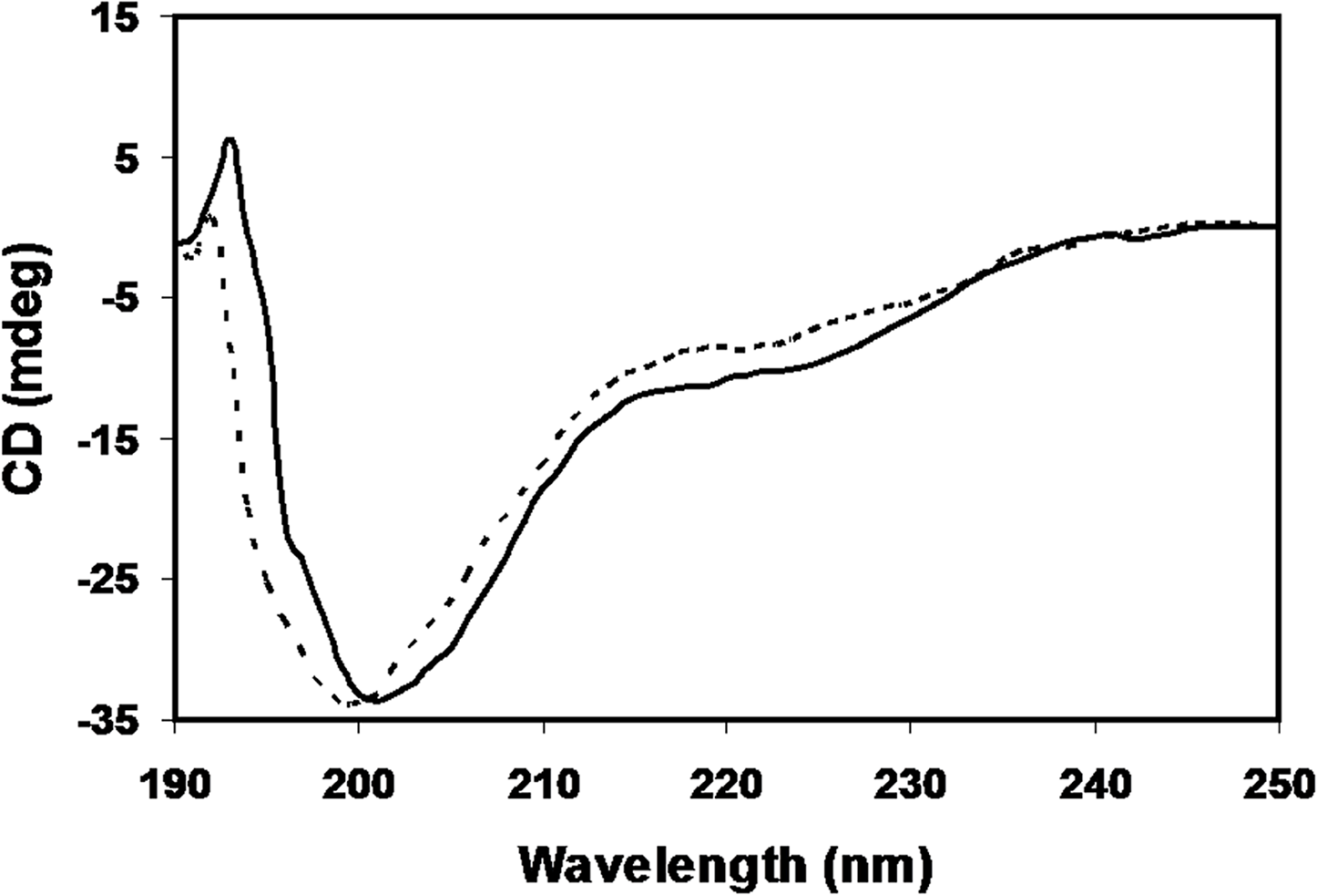
CD spectra of native histone H1 (dotted line), and histone H1 modified with 7.5 mM methylglyoxal (thick line) in far UV region (200–250 nm).

CD spectral analysis showed visible difference in molar ellipticities of native and modified histone at three wavelengths viz., 190 nm, 201 nm and 222 nm. We observed an increase in positive ellipticity at 190 nm, a decrease in negative ellipticity at 200 nm and an increase in the negative ellipticity at 222 nm. The observed ellipticity at 190 nm for native histone and methylglyoxal modified H1 was 0.909 mdeg and 1.249 mdeg respectively. The negative ellipticity was obtained at 200 nm and 222 nm. At 200 nm CD (θ) values for native histone and methylglyoxal modified H1 were – 33.615 mdeg and - 33.132 mdeg and at 222 nm the values were -8.39 and -10.2 respectively. The results are shown in Fig 7.

### Thermal Denaturation Studies (Tm) by Far-UV CD

**Fig 8:**
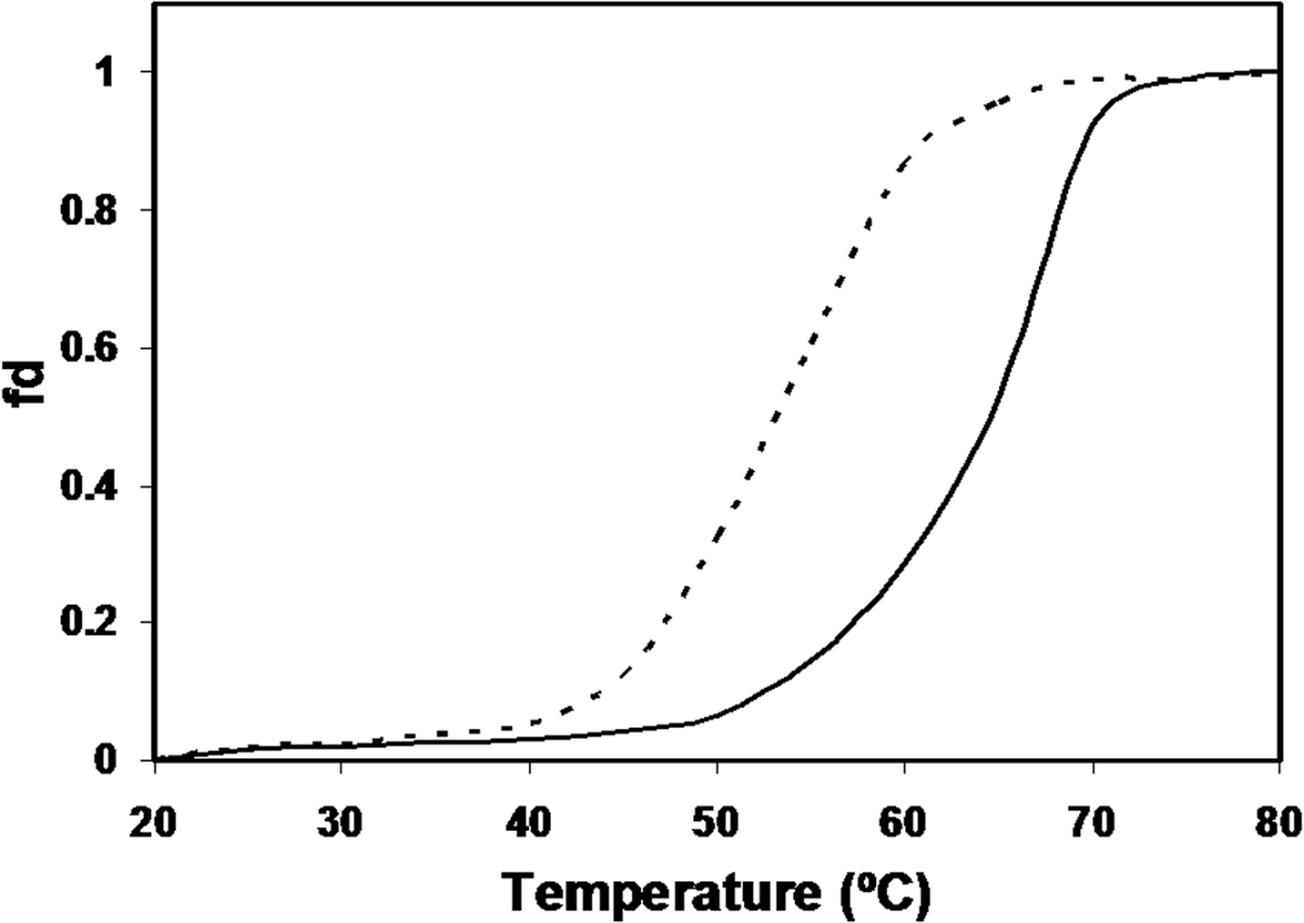
Temperature-induced thermal unfolding profile showing changes in ellipticity at 222 nm for native histone H1 (dotted line) and MG modified histone H1 (thick line).

The changes in the secondary structural characteristics obtained through melting temperature studies by following the unfolding patterns of the proteins through loss in ellipticity at 222 nm, showed early helix opening in the native histone as compared to the MG modified histone. The mid-point melting temperature (Tm) of native and MG modified histone H1 came out to be 53°C and 64°C respectively. The results are given in Fig 8.

### FTIR-ATR spectroscopic analysis

**Fig 9:**
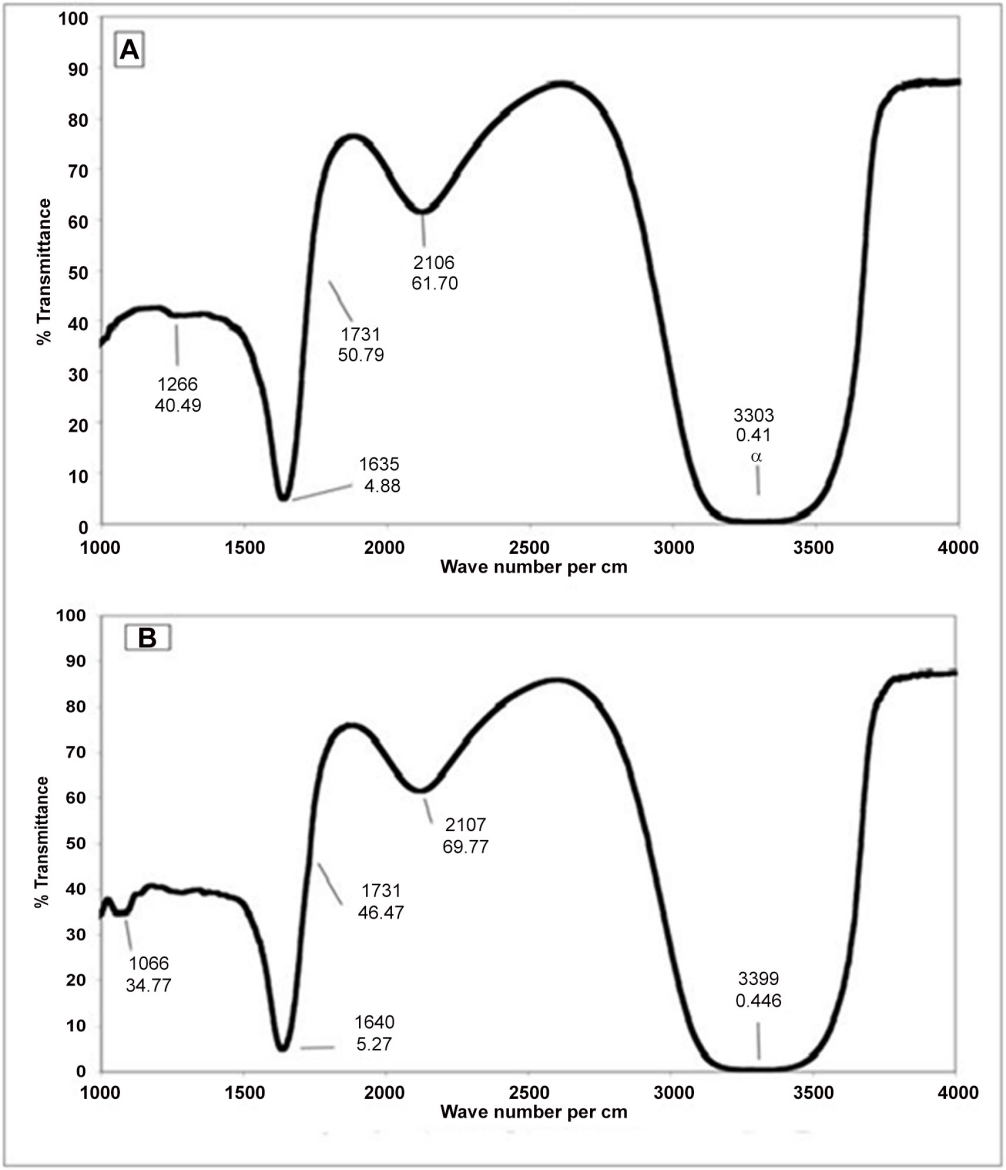
FTIR-ATR spectroscopic analysis of native (A) and MG modified histone H1 (B) recorded between 1000 cm^-1^ and 3500 cm^-1^.

FTIR results of native histone H1 showed vibrational stretching of C = O, a characteristic amide I band at 1635 cm ^-1^. However, post modification, the amide I band got shifted to 1640 cm ^-1^. Though there appeared no deep bands in amide II region, yet the difference of percent transmittance in this region was visible. We recorded less transmittance for native histone as compared to its modified form. Furthermore, we observed an additional group in the modified protein which showed a stretching band at 1731 cm ^-1^. This stretching was not observed in the native form. The modified histone also exhibited an additional band at 1066 cm ^-1^, which was absent in the native form in this region. Large bands were observed in the region with wave numbers between 3000 cm ^-1^ to 3500 cm ^-1^. This region was broader for modified histone than for the native protein. The results are shown in Fig 9.

### Liquid chromatography mass spectrometry

**Fig 10:**
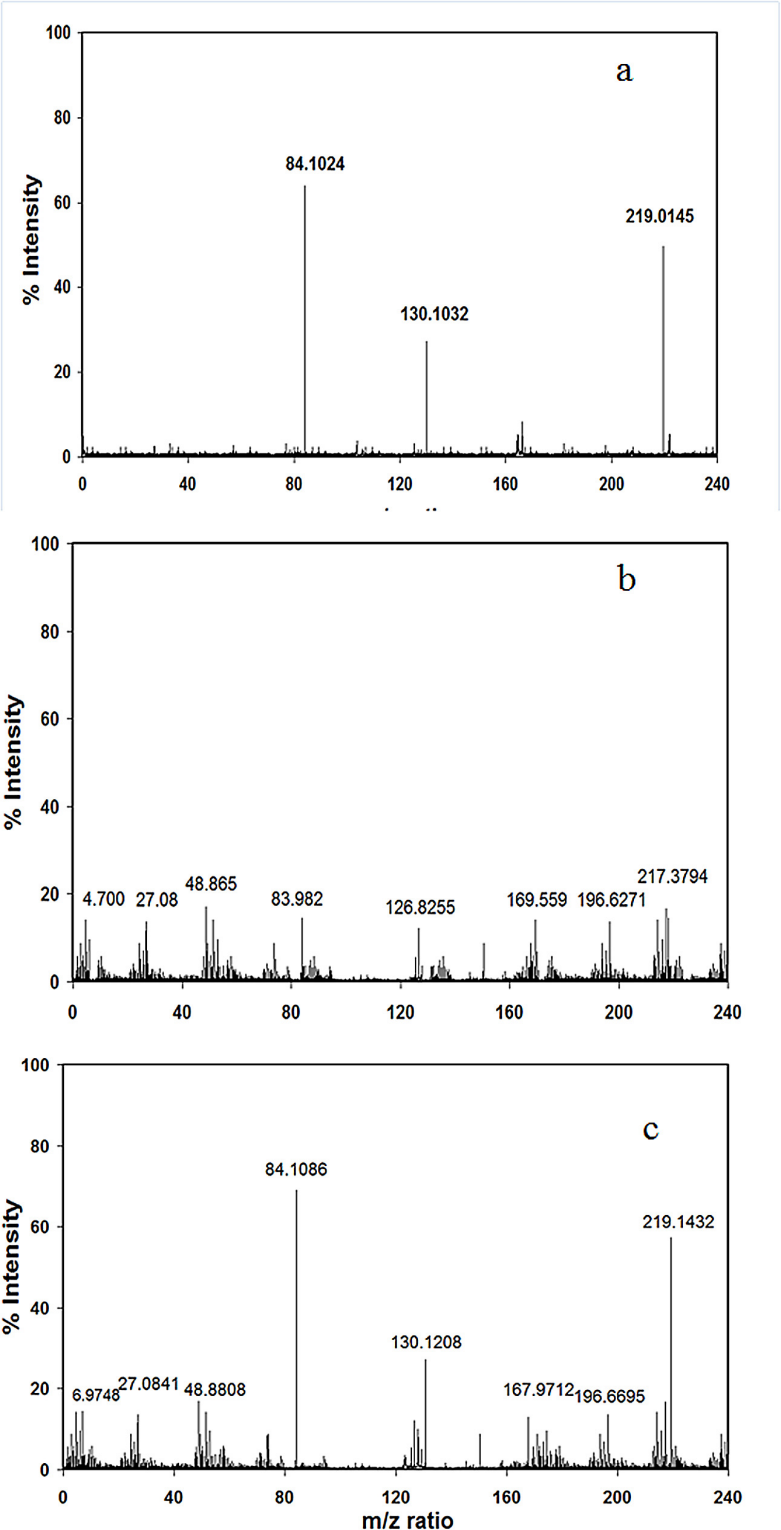
LCMS spectra of standard CEL(a) native histone H1(b) and MG modified histone H1(c).

Mass spectra of standard CEL was taken for investigating the presence of CEL and its collision-induced decomposed forms in the native and modified histones. The base peak and two product-ion mass spectra of standard CEL were observed at m/z values 219.0145, 84.1024 and 130.1032 respectively (Fig 10A). None of these peaks as observed in the standard CEL was found in case of native histone H1 (Fig 10B); the modified histone H1 gave the most prominent ion product at m/z of 84.1086. The less intense peaks were observed at m/z of 130.1208 and 219.1432 (Fig 10C).

### Scanning Electron Microscopy (SEM)

**Fig 11:**
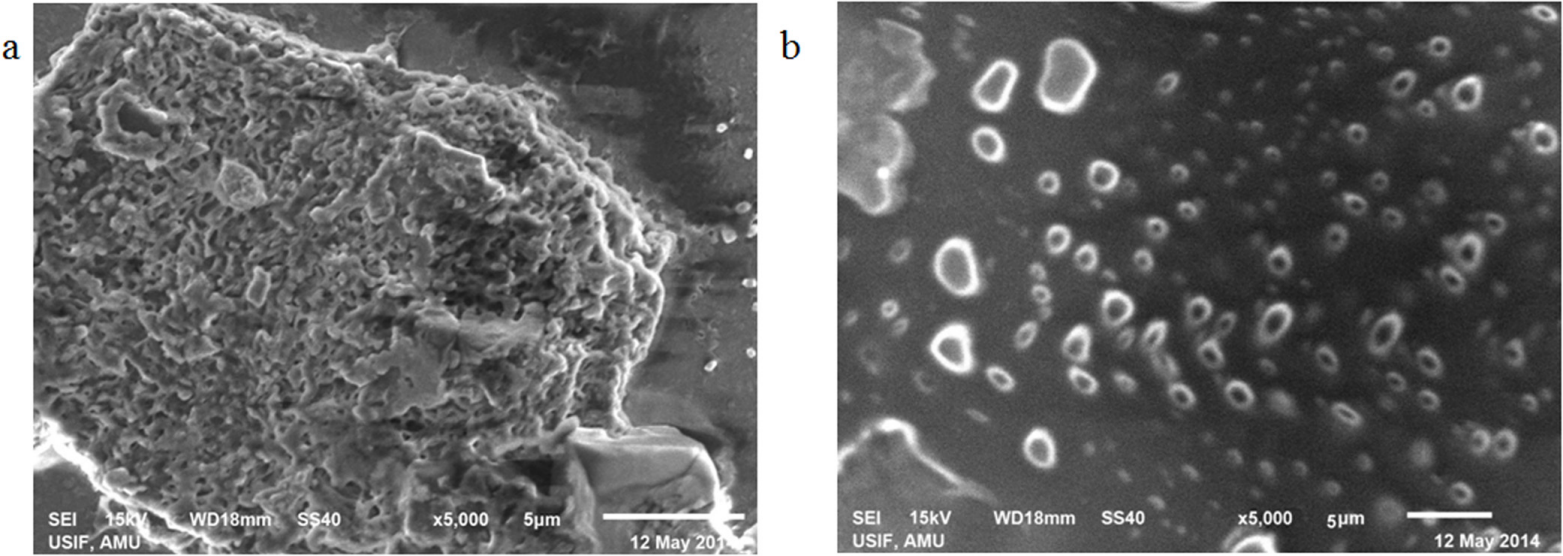
SEM micrograph of MG modified histone H1 (a) of native histone H1 (b) with image scale bar of 5 μm viewed at 5000×.

Scanning Electron Microscopy has been widely used for revealing high-resolution structural details of proteins. As observed in the SEM images, aggregate formation is clear due to modification of histone H1 by MG (Fig 11A). Native histone appears quite different from its modified form (Fig 11B).

### CD results of DNA-Protein interactions

**Fig 12:**
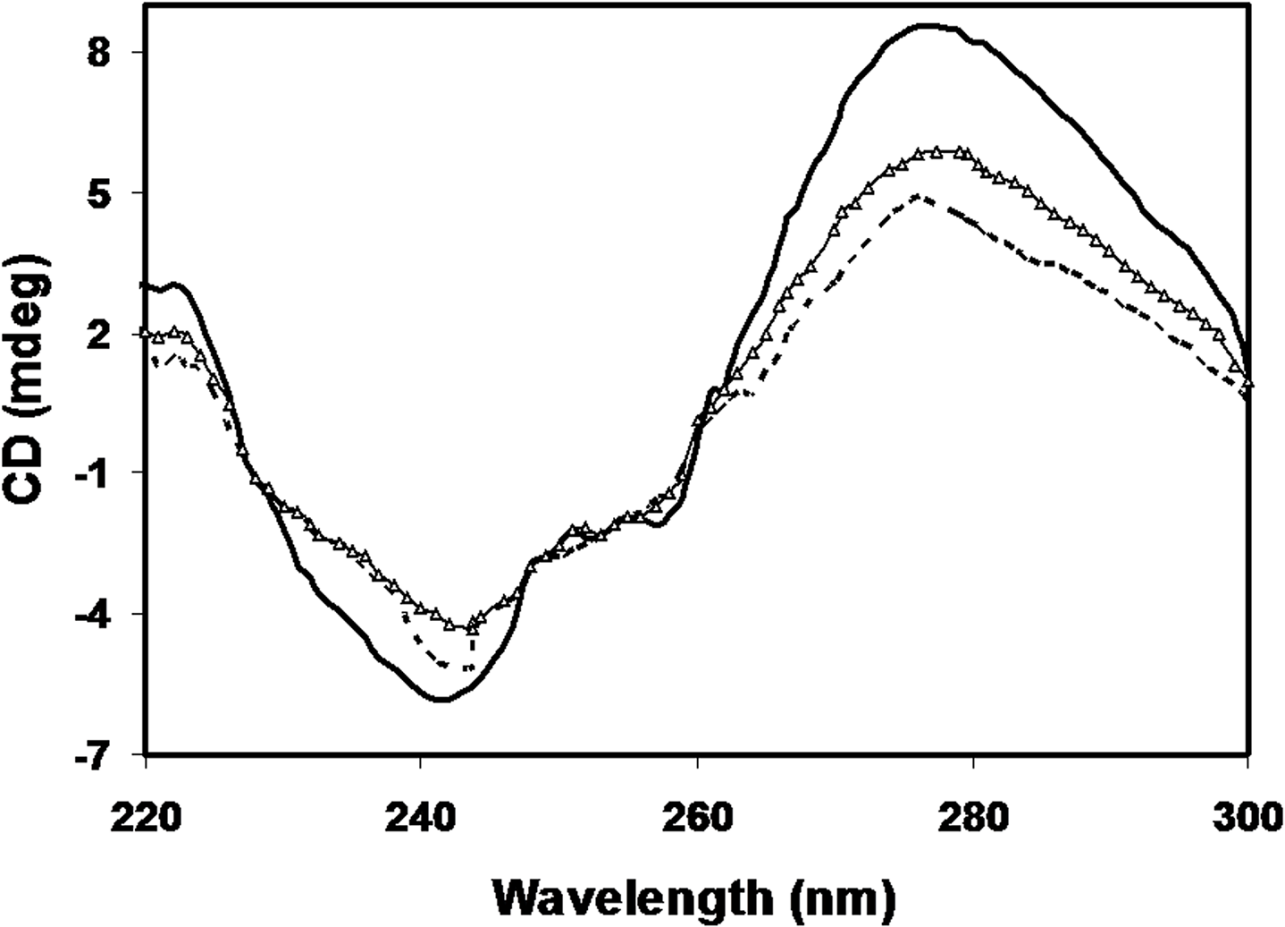
CD spectra of native histone H1 with DNA (-----), MG modified H1 with DNA (-∆-∆-) and native DNA (─) in far UV region (220–300 nm).

The circular dichroic analysis of interactions between histone H1 and DNA showed marked variations in spectral properties of native histone, its modified form and the native DNA. Native DNA showed a positive band at 277 nm (θ = 8.542) and a negative band at 243nm (θ = 5.851). The interactions between histone H1 with DNA lead to decrease in positive peak at 276 (θ = 4.9123) and an increase in the negative peak at 245 (θ = 5.092). The results of MG modified histone and DNA were different from DNA and H1-DNA as it gave intermediate peak at 277 (θ = 5.882) and an increase in the negative peak at 243 (θ = 4.394) respectively. The results are shown in Fig 12.

### Western blotting studies

**Fig 13:**
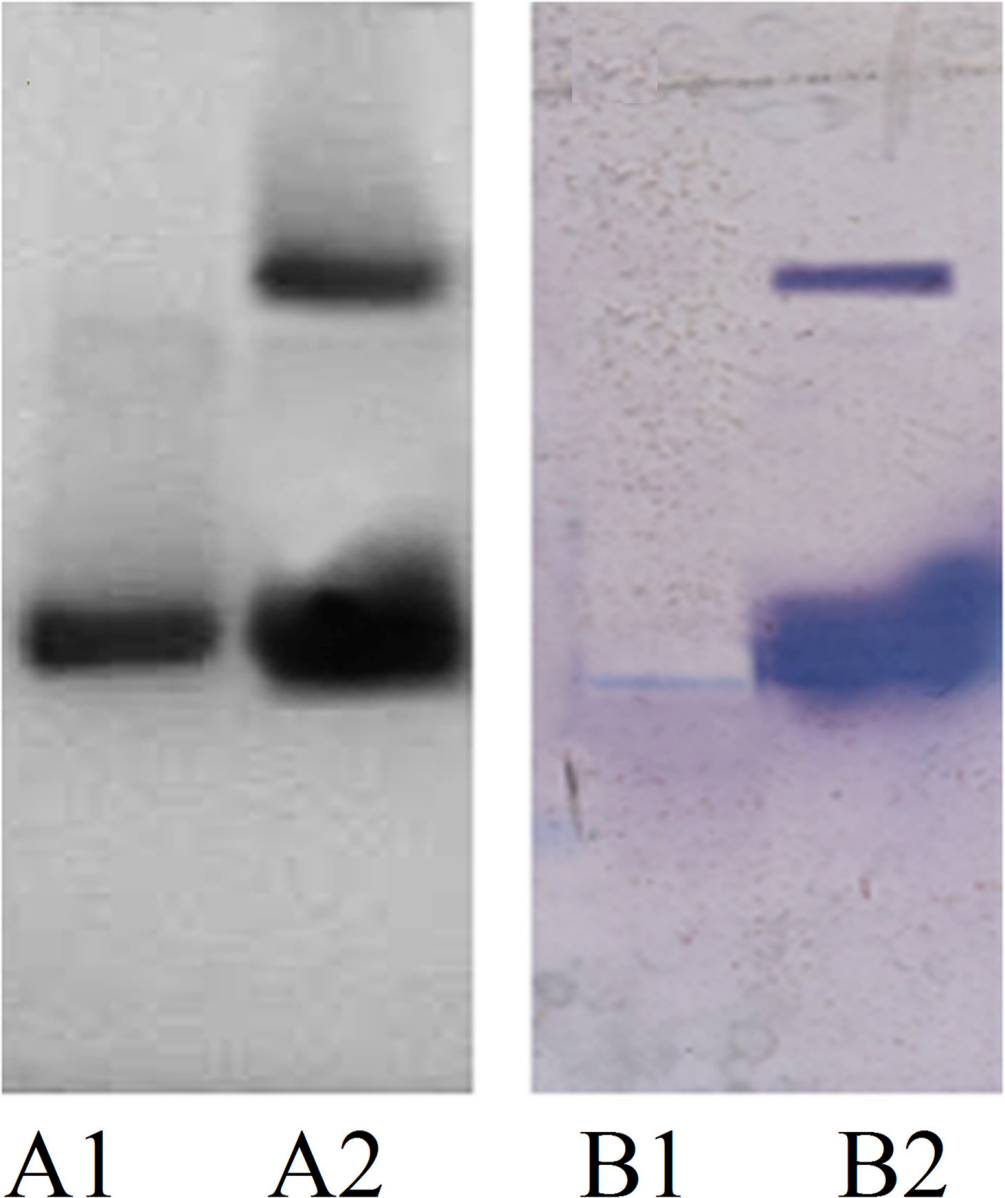
Immunoblot showing binding of anti-MG-H1antibodies induced in rabbits with native and MG-H1. A1 and A2 representSDS PAGE of native and MG-H1 respectively. B1 and B2 are the immunoblots depicting binding of anti-MG-H1 antibodies to native H1 and MG-H1 respectively.

25 μg of native and MG-H1 appeared as clear bands on polyacrylamide gel after silver staining. The modified protein showed band stretching, formation of higher molecular weight entity and an increased brightness in comparison to the native counterpart. In imunoblot analysis, antibodies induced against MG-H1 showed specific binding for the immunogen with little recognition for the epitopes on the unmodified histone H1. This reiterates our ELSIA results. The result is given in Fig 13.

### Binding of serum autoantibodies in cancer patients to native H1 and MG modified histone H1

**Fig 14:**
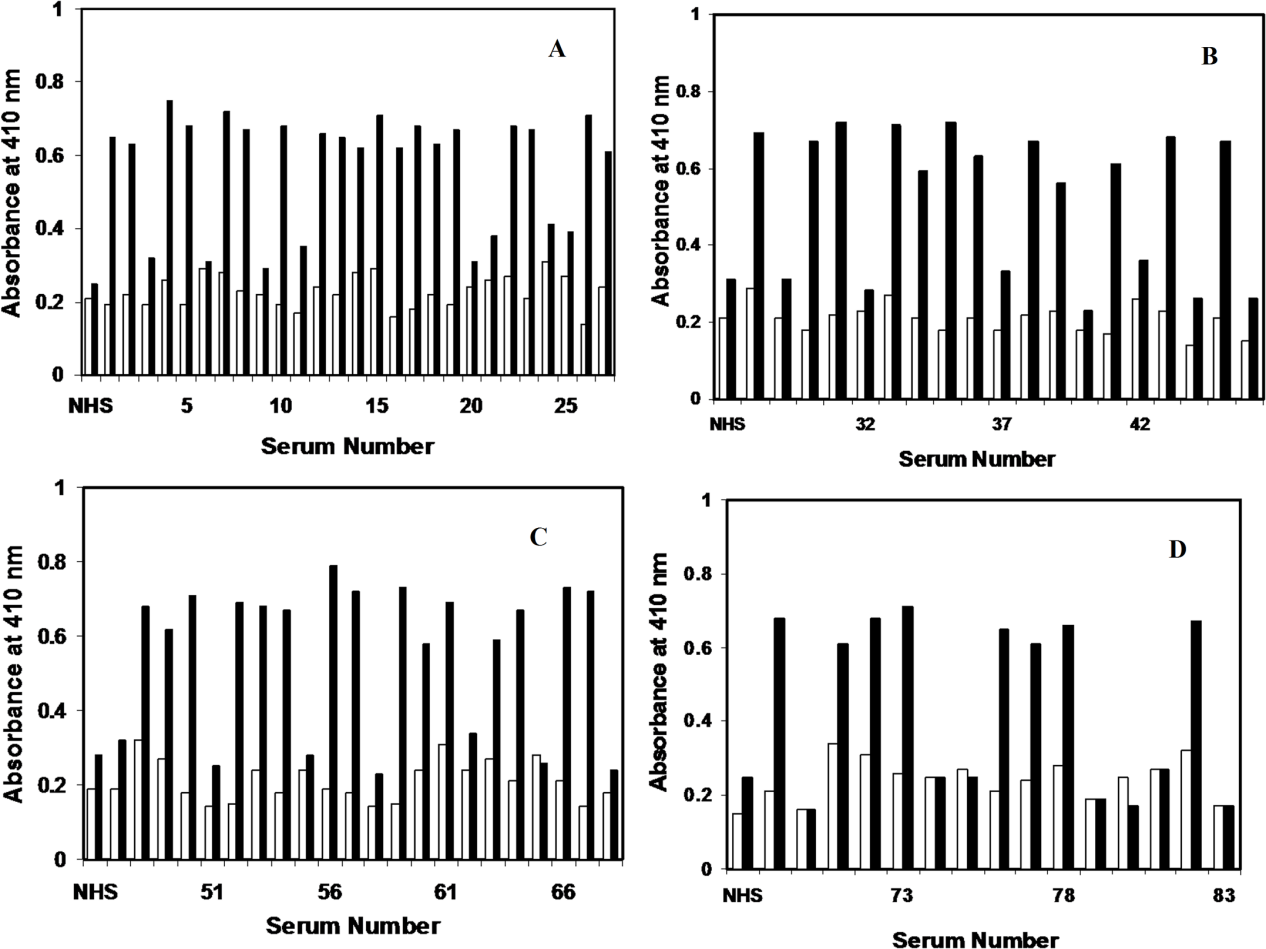
Binding of serum autoantibodies in cancer patients to native H1 and MG modified histone H1. (A) Binding of serum autoantibodies in lung cancer patients (sera no. 1–27) to native (□) and MG modified histone H1 (■). Normal human sera (NHS) served as negative control. The histogram shows mean absorbance values.(B) Binding of serum autoantibodies in prostate cancer patients (sera no. 28–46) to native (□) and MG modified histone H1 (■). Normal human sera (NHS) served as negative control. The histogram shows mean absorbance values.(C) Binding of serum autoantibodies in breast cancer patients (sera no. 47–68) to native (□) and MG modified histone H1 (■). Normal human sera (NHS) served as negative control. The histogram shows mean absorbance values.(D) Binding of serum autoantibodies in head and neck cancer patient (sera no. 69–83) to native (□) and MG modified histone H1 (■). Normal human sera (NHS) served as negative control. The histogram shows mean absorbance values.

Significantly higher percentage of serum autoantibodies from all cancer types showed enhanced binding with methylglyoxal modified H1 as compared to the native form in the direct binding ELISA experiments. Out of the total 83 serum samples, 54 samples (65.06%) exhibited appreciably higher binding to the MG-H1as against its native counterpart, thus showing better recognition for the modified histone H1. These include 19 samples of lung cancer, 12 samples of prostate cancer, 15 samples of breast cancer and 8 samples ofhead and neck cancer. The results are presented in Fig 14A–14D.

### Binding of IgG from cancer patients to native H1 and MG modified histone H1

To further evaluate the fine specificity of autoantibodies in cancer patients, IgG was isolated from serum samples showing higher binding towards MG modified histone H1 and evaluated by inhibition ELISA. IgG was mixed with varying amounts of native and MG-H1 (0–20 μg/ml) and incubated for 2 hr at 37°C and overnight at 4°C. The observed antibody inhibition fornative H1 and MG-H1 histones was in the range of 19.4%–36.2% and 49.2%–76.5% respectively.The mean percent inhibition by native H1 in the binding of autoantibodies from lung, prostate, breast, and head and neck cancers was 27.82±8.38%, 29.49±7.61%, 27.2±5.8%, and 24.51±9.5% respectively; however when MG-H1 was used as an inhibitor, the antibody activity was inhibited by 68.3±8.6%, 64.1±5.6%, 65.8±5.7%, and 52.7±7.6 respectively. Data are presented in the Table 1.

**Table 1:** Competitive inhibition of IgG isolated from sera of cancer patients with native and MG modified H1.

Type of Cancer	Sample Numbers	Max. percent inhibition		Mean±SD	
		Native H1	MG-H1	Native H1	MG-H1
**Lung cancer**	1,2,4,5,7,8,10,12,13,14,15,16,17,18,19,22,23,26,27	24.3,23.5,31.1,32.3,28.8,27.2,34.3,27.8,36.2,24.2,26.3,27.1,27.3,20.8,23.9,26.9,28.3,31.5,26.9	73.2,69.5,69.7,76.5,71.8,65.9,68.3,68.6,76.1,63.8,71.1,67.5,70.2,65.4,66.3,59.7, 61.5,72.4,61.8	27.82±8.38	68.3±8.6
**Prostate cancer**	28,30,31,33,34,35,36,38,39,41,43,45	28.5,37.1, 22.5,22.5, 34.9,27.8, 35.1,28.9, 31.2,24.3, 32.6,28.5	68.161.4, 68.9,55.2, 68.7,52.2, 62.7,66.3, 69.7,63.7, 62.9,69.4	29.49±7.61	64.1±5.6
**Breast cancer**	48,49,50,52,53,54,56,57,59,60,61,63,64,66,67	24.1,28.6,28.8,27.3,29.5,27.2,21.4,29.5,32.4,24.6,24.8,29.8,26.8,26.2,27.4	61.3,64.5,66.4,71.5,65.3,65.4,68.1,65.4,71.2,61.2,68.1,64.2,65.863.7,65.3	27.2±5.8	65.8±5.7
**Head and neck cancer**	69,71,72,73,76,77,78,82	28.9,27.1,26.4,26.7,21.3,26.1,20.2,19.4,	57.1,52.3,52.8,55.3,52.5,49.2,51.6,50.9	24.51±9.5	52.7±7.6

### Gel retardation assay

**Fig 15:**
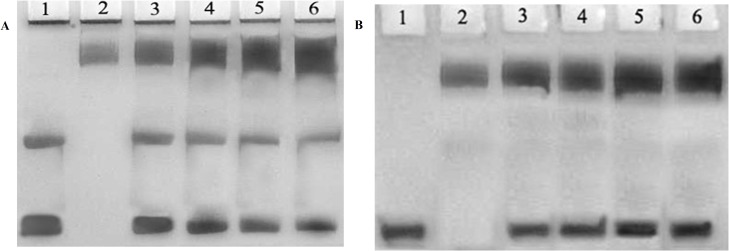
Band shift assayof IgG isolated from sera of cancer patient binding to native H1 and MG-H1. Electrophoresis was performed on 10% SDS–PAGE for 3-4 hr at 80 V. **(A)**25 μg of MG-H1 (lane 1) was incubated with 30, 40, 50 and 60 μg of anti-MG-H1 IgG (Lane 3–6). Lane 2 shows IgG alone. (**B**) 25 μg of native H1 (lane 1) was incubated with 30, 40, 50 and 60 μg of anti-MG-H1 IgG (Lane 3–6). Lane 2 shows IgG alone.

Interaction of affinity purified IgG from cancer patients with native and MG modified H1 was also studied by gel retardation assay. With increasing concentration of the IgG, formation of high molecular weight species was observed along with a decrease in unbound antigen in case of MG modified H1 (Fig 15A). However, native H1 incubated with the IgG showed weak interaction and that too at higher concentrations of IgG (Fig 15B).

## Discussion

Cancer being one of the deadliest diseases causing millions of causalities across the globe has led the researchers to focus on the early detection strategies of the disease. In this regard, post translational modifications of the proteins are expected to yield new and more informative targets, for the development of simple non-invasive screening methods for the detection of cancer at the earlier stages [2].Hyperglycemiais being described as the molecular link between diabetes and canceras it induces oxidative stress in a variety of cells via various metabolic pathways leading to oxidative damage of DNA and proteins, an initial step of carcinogenesis. AGEs and their receptors (RAGE) have been implicated in cancer development and the involvement of the AGE/RAGE axis in the cancer progression has also been reported[5, 9, 10,31]. Higher levels of CML-AGE and lower levels of sRAGE have been associated with a greater risk of pancreatic, oral and colorectal cancer. The importance of RAGE in the early pathogenesis of Kras-driven pancreatic cancer has also been studied [7, 11, 32–34]. Furthermore, histone modifications are known to play a necessary functional role in the regulation of processes like gene transcription and in the proliferation, metastasis, chemotherapy and other aspects of human cancers [16]. Among the histones, histone H1, the linker protein that serves the most important role in chromatin condensation, protection of DNA from external damage and in activation and repression of specific genes is glycated by glucose, fructose, ribose, and ADP-ribose leading to the formation of by-products like pentosidine, argpyrimidine etc. [35–36]. However, the role of methylglyoxal, an oxo-aldehyde, which is a very strong glycating agent with involvement in the modification of a variety of proteins in various pathological disorders, is yet to be ascertained. Also, MG mediated modifications may lead to alterations in the immunogenicity of histone H1 and mayhave possible implications in the generation of an auto-immune response in the various types of cancers. To study the same, we designed a study on H1 modifications by MG and its role in the generation of neo-epitopes and consequent autoimmune response in cancers.

Hyperchromicities in the UV spectra of modified histones points towards their structural damage induced by modification of the aromatic residues or the variations in their microenvironment and the hump like peaks at 340 nm in the modified histones correspond to generation of advanced glycation end products [37].

The gradual decrease in the intrinsic fluorescence of histones upon modification shows MG mediated destruction or modification of tyrosine microenvironment of histones which may be a due to transition from a random coil conformation to a more orderly state and by the effect of methylglyoxal as a quenching agent. The decline in the fluorescence emission maximum (λm) upon glycation is well known [18, 38] etc. The red shift of 3, 6, 8 and 10 nm in the emission wavelength reflect the exposure of the tyrosine chromophores to the solvent as well as interaction with the quenching agent. Furthermore, additional hump like peaks observed in the modified protein at 440 nm correspond to the generation of AGEs[39].

The increase in the electrophoretic mobility of methylglyoxal modified histones in comparison to native histone H1 shows a progressive loss of positive charges of epsilon amino groups of lysine residues in MG modified histones. Large sized molecules that are visible at higher molecular weight positions in the gel correspond to the formation of dimers in the modified proteins and are likely the products of intermolecular cross-linking. Moreover, higher molecular weight aggregates (molecular weight above 200 kDa) that did not enter the gel were also observed in the proteins incubated with methylglyoxal. The band intensities exhibited a proportional increase with the increasing concentrations of modifying agent showing encapsulation of positive charges of lysine.Glycation induced aggregate formation and cross links in various proteins has been reported earlier [40–41]. Furthermore, a study of histone H1 has earlier shown the formation of cross linked AGE protein adducts in the in-vivo modified histone H1 as indicative of protein damage [42].Our results show that the di-carbonyl induced aggregation in histone H1 may influence the histone-DNA interaction and affect the chromatin integrity.

The substantial increase in the generation of AGEs as determined by 74% increase in AGE fluorescence intensity in case of MG modified histone H1shows the availability of lysine and arginine residues of histones to dicarbonyls for modification under oxidative stress. The expected AGE product formation in this case was N-epsilon-(*carboxyethyl*)*lysine*, which was later confirmed by FTIR spectroscopy and LCMS. Earlier reports show the formation of histone-histone cross-links and fluorescent AGEs in histones by ADP-ribose, glucose, fructose and ribose [36]. It appears that glycation has a wider impact across the spectrum of extra cellular, intra cellular and nuclear proteins, however;histone glycation is more significant because any structural changes in the characteristics of histone proteins might have serious impact on the modulation of chromatin structure and the regulation of gene expression. The generation of AGEs is pathologically significant and their generation in histones may have a wider impact.

Glycation affects the surface properties of proteins by altering their overall surface charge; and here in this case, MG-H1 caused a significant decrease of 79.9% in ANS florescence intensity in comparison to native H1, pointing towards the reduction of hydrophobic clusters in the protein due to the introduction of alpha and beta structure upon modification. There appears a transition from random coil conformation of the native histone to its more organised form in the modified state leading to the masking of hydrophobic patches in histone H1. The hypsochromic shift in the fluorescence of the modified histone is due to the interaction of the charged group of lysine and arginine with the sulfonate group of ANS. There is an intermolecular charge transfer of a positive charge near the-NH group of ANS that leads to the blue shift of ANS fluorescence [43].

Protein carbonyls, the general markers for protein oxidation have been attributed with important role in various pathological conditions including rheumatoid arthritis, adult respiratory syndrome pulmonary fibrosis, diabetes, Parkinson's disease, systemic lupus erythematosus, cystic fibrosis and Alzheimer's disease [44]. The substantial increase in the carbonyl content of MG-H1 against its native counterpart shows an aggressive oxidation by methylglyoxal, that can be attributed to glycation prone amino acids like lysine, arginine etc, that are available to reducing sugars for glyco-oxidation. ADP ribose has been earlier implicated in in-vivo carbonylation of histone H1 with deleterious effects on chromatin structure and function [45], MG could be potentially more dangerous because of its strong glycating potential.

Histones are dominantly of helical structure with α-helices accounting for 65–70% of the total structure, 3% of the residues have been assigned to short parallel β-sheets and the remainder is said to be disordered. However in water, the random coil structure dominates. Certain secondary structure inducing agents like trifluoroethanol have been shown to induce alpha helix in the histone protein. Under saline conditions ordered structure has been observed due to induction of β structure [46]. In case of the modified histone H1, CD spectra presented marked variations with hardly any regions of overlap with its native counterpart. The observed enhanced negative ellipticity at 222 nm in the modified histone against the native histone shows the formation of more ordered secondary structure and increased compactness. The increase in mean residual molar ellipticity values from -8.39 mdeg in native histone to -10.2 mdeg in the modified protein amounts to 12% increase in alpha helix. The increase in ellipticity at 190 nm from 0.909 mdeg for native histone to 1.249 mdeg for the modified histone shows induction of beta structure and enhanced folding in the modified form. The increased alpha and beta structures in the protein, upon modification, are compensated by the loss of random coil content which is obvious from the decrease in negative ellipticity at 200 nm. Methylglyoxal induced glycation is known to induce ordered structures in many proteins like haemoglobin, myoglobin, α-crystallin, insulin and histone H2A [18].

Thermal denaturation studies revealed thermal stability of the modified histone, which further confirms the ordered structure produced after incubation with methylglyoxal. Earlier, we have shown enhanced thermodynamic stability in case of modified histone H2A wherein methylglyoxal-mediated cross-linking and increase in hydrogen bonding led to increase in melting temperature (Tm) and the enthalpy change (∆H) in DSC measurements. The increase in melting temperature in the glycated proteins has been reported earlier [18].

In the FTIR analysis, shifting of bands to different wave numbers, changed transmittance and the generation of the new bands was observed upon glycation. Amide I band shifted from 1639 cm^-1^ to 1635 cm^-1^ showing a transition within the beta conformation of histone upon modification. In amide II region, although no deep bands appear, but the analysis of the percent transmittance shows changes in the protein structure. The reaction of methylglyoxal with lysine and arginine residues of histone H1 generates a new band in the modified histone at 1731 cm^-1^which corresponds to a carboxyethyl group exhibiting a typical C = O stretching, thus showing the formation of carboxyethyllysine, in conformity with an earlier report [47]. The band appearing at 1066 cm^-1^ in modified histone shows a characteristic N-Cα vibration in polypeptides and a marker to the protein glycosylation [48].The large band observed in 3000 cm^-1^ and 3500 cm^-1^ with broadened band for the modified histone shows N-H and O-H stretching and the accumulation of OH groups by sugar moiety in the modified histone [18].

Mass spectrometry showed the generation of base peaks and two product-ion mass spectra in the MG modified histone H1 similar to that of standard CEL thus confirming the generation of carboxyethylated lysine residues and their decomposition forms by the applied collision energy. Absence of such peaks in their native counterpart shows the absence of carboxyethylated lysine residues in the unmodified form. Of the three peaks observed in H1 histone, the most prominent ion product appeared at m/z 84.1086 with lesser peaks at m/z 130.1208 and 219.1432. The higher intensity of peak at 84.1086 shows more specific mass transitions of m/z 219.1432→84.1086 than to the mass transitions of m/z 219.1432→130.1208. Earlier studies using mass spectrometry have shown that lysine residues of proteins are potential targets of dicarbonyl compounds’ leading to the generation of a number of adducts, like imidazolium cross-link structure, imidazolysine, CEL [49].An earlier study, using mass spectrometry for characterization of modifications in histones after incubation with glucose in pseudo-physiological conditions, has reported 14 possible glycation sites in histone H1 and 6 possible glycation sites in histone H2B. They suggest that the glycated lysines can vary from molecule to molecule [50].Previously, freshly isolated calf thymus histone H1 has been studied to develop procedures for histone extraction, isolation and quantification of CML, immunoblotting of adducts like CML and argpyrimidine, and to perform tandem mass spectroscopic analysis of glycated histones, and it has been revealed that carboxymethylation specific sites are present in the N-terminal region of the sequence of histone H1 close to the globular domain[42].Our study show accumulation of methylgyoxal residues on the amino acids residues of histone H1 and confirms the structural alterations due to CEL adduct formation in histone H1. We have hypothesised and also evaluated the role / implications of CEL in the generation of antibodies in various cancer types.

The micro-architectural details and morphological characteristics of glycated proteins as studied by scanning electron microscopy have shown the formation of different types of aggregates [26]. MG mediated modifications led to the formation of large amorphous aggregate structure against the small granular shape like structures in the native histone H1. These amorphous aggregates could have a pathological significance as earlier studies have shown amorphous aggregates to be cytotoxic in comparison to the fibrillar morphology [51]. Earlier as well, globular and amorphous aggregates have been reported in human serum albumin [52]. In another study, glycation of hen egg white lysozyme by three different sugars was found to induce amorphous and globular morphology with enhanced β-sheet content [53]. SEM studies have also shown the amorphous structure of haemoglobin aggregates after glycation by 70% glyoxal and presence of branched fibrils like aggregates after prolonged glycation of haemoglobin by 30% glyoxal [26]. Furthermore, glycation of albumin by glucose has been reported to form irregularly shaped sheets with varying sizes, together with free and clustered granular precipitates. Glycation with glyoxylic acid has been found to form large branched chains of globular aggregates in albumin. Glyceraldehyde modified albumin was found to appear as clustered granules with sporadically occurring linear rods and fructose modification led to large and condensed amorphous aggregates [54]. It appears from our observation and the earlier reports that proteins acquire diverse types of shapes after glycation and these variable shapes may have some pathological relevance.

Furthermore, we report a difference in DNA binding properties of histone H1 due to the changes in its conformational properties upon modification. Methylglyoxal mediated modification of H1 has changed the characteristic cooperative binding to DNA and a stronger condensation of modified histones to DNA. The interactions between histone proteins and the DNA led to the decrease in ellipticity in positive peak at 276 nm and an increase in the ellipticity in the negative peak at 245 nm. Earlier works have also reported similar CD spectra as a consequence of histone-DNA interactions. The new observation was the intermediate band for MG modified histone H1. The intermediate spectrum points towards the structural damage to the protein leading to decreased interactions of histones with the DNA. The observed intermediate spectrum may be a consequence of the presence of mixtures of bound and free DNA in solution due to incomplete complex formation between modified histone H1 and DNA. The results confirm that the glycated histones behave differently from their native forms. Earlier, the accumulation of AGEs was found directly related to DNA damage in hepatocytes of rats by X-ray irradiation studies [55]. Furthermore, AlF4, an aluminium derivative, leads to glyoxidation of histone H1 in the nucleotide-binding sites, and has been associated with pathogenesis of aluminium-induced encephalopathy and Alzheimer's disease [56]. It shows the influence of MG on the binding patterns of histone H1 and DNA, with a consequential influence on chromatin structure and having possible pathological implications.

Western blot analysis presented a conclusive proof for the specific antibody response against MG-H1. The immunoblot showed a strong antibody reaction towards the MG modified histone H1 as compared to its native form. Though bands for both the native and MG modified forms of histones H1 were seen on the electrophoretic gel, only the bands for modified histone could be clearly seen on the immunoblot, thus showing that the antibodies against the modified histone are highly specific towards the immunogen than the native protein. It may be concluded that MG induced modification has generated neo epitopes on histones H1 that induce high titre and specific antibodies against the same.

Out of the total 83 serum samples from cancer patients, 54 (65.06%) exhibited appreciably higher binding with MG-H1as against its native counterpart, thus exhibiting better recognition for the modified histone H1 by cancer autoantibodies.Sera from normal human subjects did not show appreciable binding with histones H1 in either of its native or modified form. Better recognition of MG-H1 by cancer autoantibodies in various cancer types points towards the presence of an auto-antibody population against MG modified histones in the sera of cancer patients.

The competitive inhibition studies data, wherein substantially higher inhibition of cancer auto-antibodies activity was observed with MG-H1, proves the specificity of these antibodies towards the modified epitopes on histone protein.

The solid phase immunoassay results were further confirmed by gel shift assay which showed the formation of high molecular weight immune complexes between cancer IgGs and modified histone H1. These results point towards the generation of auto-antibodies in cancer patients against the modified epitopes of histone H1.

The presence of autoantibodies in cancer has become much relevant in recent years. Some workers demonstrated that autoantibodies purified from the sera of breast cancer patients activate muscarinic acetylcholine receptors in tumor cells. Immunoglobulin G (IgG) from breast cancer patients mimics the action of the muscarinic agonist carbachol stimulating MCF-7 cell proliferation, migration and invasion. These autoantibodies represent a previously unaddressed source of sensitive biomarkers for early detection of cancer [1–2]. So, we believe that further studies on auto-antibodies against the modified epitopes on histone H1 may pave the way for the development of a diagnostic immuno-marker.

It may be added that the immunological response against modified epitopes of histones in autoimmune disorders has earlier been reported in systemic lupus erythematosus and diabetes type 1 [57–58]. Oxidation of histone H2A proteins has been correlated with the pathogenesis of RAby stimulating T and B lymphocytes, especially T helper (Th)1 cells [59]. Our study reveals auto immune response against methylglyoxal modified histone H1 in the cancer patients and thereby broadens the understanding of histone autoimmunity for further research.

## Conclusion

This study shows that methylglyoxal induces gross structural changes in the linker histone H1 resulting in altered biophysical and biochemical characteristics. Such structural perturbations generate neo-epitopes on the modified histone H1, generating a highly specific immune response, and leading to the induction and elevated levels of circulating antibodies against the modified histones. Since research for early detection of cancer is now being focussed on the proteins with post translational modifications, that are immunogenic and stimulate cellular and humoral immune responses, the methylglyoxal modified histones may also be considered as potential antigenic candidates for eliciting autoimmune response in cancer patients.

## References

[1] Wandall HH, Blixt O, Tarp MA, Pedersen JW, Bennett EP, Mandel U, et al Cancer biomarkers defined by autoantibody signatures to aberrant O-glycopeptide epitopes. Cancer Res. 2010; 70: 4: 1306–13. 10.1158/0008-5472.CAN-09-2893 20124478 PMC5538776

[2] Pedersen JW, Wandall HH. Autoantibodies as Biomarkers in Cancer. Lab Medicine. 2011; 42: 623–628.

[3] Anderson KS, Labaer J. The sentinel within: exploiting the immune system for cancer biomarkers, J Proteome Res. 2005; 4: 1123–33. 16083262 10.1021/pr0500814 PMC2522321

[4] Lu H, Goodell V, Disis ML. Humoral immunity directed against tumor-associated antigens as potential biomarkers for the early diagnosis of cancer. J Proteome Res. 2008; 7: 1388–94. 10.1021/pr700818f 18311901

[5] Abe R, Yamagishi S. AGE-RAGE system and carcinogenesis.Curr Pharm Des. 2008; 14: 10: 940–5. 18473843 10.2174/138161208784139765

[6] van Heijst JW, Niessen HW, Hoekman K, Schalkwijk CG. Advanced glycation end products in human cancer tissues: detection of Nepsilon-(carboxymethyl)lysine and argpyrimidine. Ann N Y Acad Sci. 2005; 1043: 725–733. 16037299 10.1196/annals.1333.084

[7] Ko SY, Ko HA, Shieh TM, Chang WC, Chen HI, Chang SS, Lin IH. Cell migration is regulated by AGE-RAGE interaction in human oral cancer cells in vitro. PLoS One. 2014; 9:10: e110542 10.1371/journal.pone.0110542 25330185 PMC4199749

[8] Hsu PP, Sabatini DM. Cancer cell metabolism: Warburg and beyond. Cell. 2008; 134: 703–707. 10.1016/j.cell.2008.08.021 18775299

[9] Singh R, Barden A, Mori T, Beilin L. Advanced glycation end-products: a review. Diabetologia. 2001; 44: 129–146. 11270668 10.1007/s001250051591

[10] Kalea AZ, Schmidt AM, Hudson BI.Alternative splicing of RAGE: roles in biology and disease. Front Biosci.2011; 17: 2756–70 10.2741/3884 21622207

[11] Kang R, Loux T, Tang D, Schapiro NE, Vernon P, Livesey KM, et al The expression of the receptor for advanced glycation endproducts (RAGE) is permissive for early pancreatic neoplasia.Proc Natl Acad Sci. USA. 2012; 109: 18:7031–6. 10.1073/pnas.1113865109 22509024 PMC3345001

[12] Giovannucci E, Harlan DM, Archer MC, Bergenstal RM, Gapstur SM, Habel LA, et al Diabetes and cancer: a consensus report. Diabetes Care. 2010; 33: 7: 1674–85. 10.2337/dc10-0666 20587728 PMC2890380

[13] Stopper H, Schinzel R, Sebekova K, Heidland A. Genotoxicity of advanced glycation end products in mammalian cells. Cancer Lett. 2003; 20:190: 2: 151–6. 10.1016/s0304-3835(02)00626-2 12565169

[14] Soussi T. p53 Antibodies in the sera of patients with various types of cancer: a review, Cancer Res. 2000; 60: 1777–1788. 10766157

[15] Chervona Y.Costa M. Histone modifications and cancer: biomarkers of prognosis? Am J Cancer Res. 2012; 2: 5: 589–97. 22957310 PMC3433108

[16] Luo XG, Guo S, Guo Y, Zhang CL. Breast Cancer—Focusing Tumor Microenvironment, Stem cells and Metastasis. Gunduz M (Ed.) 2011.

[17] Lo TW, Westwood ME, McLellan AC, Selwood T, Thornalley PJ. Binding and modification of proteins by methylglyoxal under physiological conditions.J Biol Chem. 1994; 269: 51: 32299–305. 7798230

[18] Mir AR, uddin M, Alam K, Ali A. Methylglyoxal mediated conformational changes in histone H2A-generation of carboxyethylated advanced glycation end products. Int J Biol Macromol. 2014; 69: 260–6. 10.1016/j.ijbiomac.2014.05.057 24879922

[19] Chellan P, Nagaraj R. Protein crosslinking by the Maillard reaction: dicarbonyl-derived imidazolium crosslinks in aging and diabetes. Arch Biochem Biophys. 1999; 368: 1: 98–104. 10415116 10.1006/abbi.1999.1291

[20] Roberts MJ, Wondrak GT, Laurean DC, Jacobson MK, Jacobson EL. DNA damage by carbonyl stress in human skin cells. Mutat Res. 2003; 522: 1–2: 45–56. 12517411 10.1016/s0027-5107(02)00232-4

[21] Laemilli UK. Cleavage of structural protein during the assembly of the head of bacteriophage T4. Nature. 1970; 227: 680–685. 5432063 10.1038/227680a0

[22] Cardamone M, Puri NK. Spectrofluorimetric assessment of the surface hydrophobicity of proteins. Biochemistry. 1992; 282: 589–93. 10.1042/bj2820589 PMC1130822 1546973

[23] Hawkins CL, Morgan PE, Davies MJ. Quantification of protein modification by oxidants, Free Radical Biology & Medicine. 2009; 46: 965–988. 19439229 10.1016/j.freeradbiomed.2009.01.007

[24] Correa DHA, Ramos CHI. The use of circular dichroism spectroscopy to study protein folding, form and function. African Journal of Biochemistry Research. 2009; 3: 5: 164–173.

[25] Zhang G, Huang G, Xiao L, Mitchell AE. Determination of advanced glycation endproducts by LC-MS/MS in raw and roasted almonds (Prunus dulcis). J Agric Food Chem. 2011;59: 22: 12037–46. 10.1021/jf202515k 21980977

[26] Iram A, Alam T, Khan JM, Khan TA, Khan RH. Naeem A.Molten globule of hemoglobin proceeds into aggregates and advanced glycated end products.PLoS ONE. 2013; 8:8. 10.1371/journal.pone.0072075 PMC3753358 23991043

[27] Ali R, Alam K. Evaluation of antibodies against oxygen free radical-modified DNA by ELISA. Methods Mol Biol. 2002; 186: 171–81. 12013765 10.1385/1-59259-173-6:171

[28] Shahab U, Tabrez S, Khan MS, Akhter F, Khan MS, Saeed M, et al Genotoxicity and immunogenicity of DNA-advanced glycation end products formed by methylglyoxal and lysine in presence of Cu2+. Biochem Biophys Res Commun. 2011; 407: 568–74. 10.1016/j.bbrc.2011.03.064 21420380

[29] Sambrook J, Fritsch EF, Maniatis T. Molecular cloning, A laboratory Manual 2nd ed. Cold Spring Harbor Laboratory Press: Cold Spring Harbor. NY; 1989.

[30] Ansari NA, Moinuddin, Alam K, Ali A. Preferential recognition of Amadori-rich lysine residues by serum antibodies in diabetes mellitus: role of protein glycation in the disease process. Human Immunol. 2009; 70: 417–24. 19332092 10.1016/j.humimm.2009.03.015

[31] Logsdon CD, Fuentes MK, Huang EH, Arumugam T. RAGE and RAGE ligands in cancer. Curr Mol Med. 2007; 7: 777 *–* 89. 18331236 10.2174/156652407783220697

[32] Sparvero LJ, Asafu-Adjei D, Kang R, Tang D, Amin N, Im J, et al RAGE (Receptor for Advanced Glycation Endproducts), RAGE ligands, and their role in cancer and inflammation.J Transl Med. 2009; 7:17 10.1186/1479-5876-7-17 19292913 PMC2666642

[33] Krechler T, Jáchymová M, Mestek O, Zák A, Zima T, Kalousová M. Soluble receptor for advanced glycation end-products (sRAGE) and polymorphisms of RAGE and glyoxalase I genes in patients with pancreas cancer. Clin Biochem. 2010; 43: 882–6. 10.1016/j.clinbiochem.2010.04.004 20398646

[34] Jiao L, Taylor PR, Weinstein SJ, Graubard BI, Virtamo J, Albanes D, et al Cancer, Advanced glycation end products, soluble receptor for advanced glycation end products, and risk of colorectal cancer. Epidemiol Biomarkers Prev. 2011; 20:7: 1430–8. 10.1158/1055-9965.EPI-11-0066 PMC3132292 21527578

[35] Wolffe AP, Khochbin S, Dimitrov S. What do linker histones do in chromatin? Bioessays. 1997; 19: 3: 249–255. 9080775 10.1002/bies.950190311

[36] Talasz H, Wasserer S, Puschendorf B.Non enzymatic glycation of histones in vitro and in vivo. Journal of Cellular Biochemistry. 2002; 85 24–34. 11891847

[37] Schmitt A, Schmitt J, Münch G, Gasic-Milencovic J.Characterization of advanced glycation end products for biochemical studes: side chain modifications and fluorescence characteristics. Anals Biochem. 2005; 15: 338: 2: 201–15. 10.1016/j.ab.2004.12.003 15745740

[38] Arfat MY, Ashraf JM, Arif Z, Moinuddin, Alam K. Fine characterization of glucosylated human IgG by biochemical and biophysical methods. Int J Biol. Macromol. 2014; 69: 408–15. 10.1016/j.ijbiomac.2014.05.069 24953604

[39] Kishore D, Kundu S, Kayastha AM. Thermal, Chemical and pH Induced Denaturation of a Multimeric β-Galactosidase Reveals Multiple Unfolding Pathways. PLoS One. 2012; 7: 11. 10.1371/journal.pone.0050380 PMC3503960 23185611

[40] Kang JH.Oxidative modification of human ceruloplasmin by methylglyoxal: an in vitro study. J Biochem Mol Biol. 2006; 335–8. 16756764 10.5483/bmbrep.2006.39.3.335

[41] Nagaraj RH, Shipanova IN, Faust FM. Protein cross-linking by the Maillard reaction. Isolation, characterization, and in vivo detection of a lysine-lysine cross-link derived from methylglyoxal. J Biol Chem. 1996; 271:32: 19338–19345. 8702619 10.1074/jbc.271.32.19338

[42] Pashikanti S, Boissonneault GA, Cervantes-Laurean D. Ex vivo detection of histone H1 modified with advanced glycation end products. Free Radic Biol Med. 2011; 50(10):1410–6 10.1016/j.freeradbiomed.2011.01.034 21315148

[43] Khan TA, Saleemuddin M, Naeem A. Partially Folded Glycated State of Human Serum Albumin Tends to Aggregate. Int J Pept Res Ther. 2011; 17: 2011: 271–279.

[44] Dalle-Donne I. Aldini G, Carini M, Colombo R, Rossi R, Milzani A. Protein carbonylation, cellular dysfunction, and disease progression. J Cell Mol Med. 2006; 10: 389–406. 16796807 10.1111/j.1582-4934.2006.tb00407.x PMC3933129

[45] Wondrak GT, Cervantes-Laueant, Jacobson EL, Jacobson MK. Histone carbonylation in vivo and in vitro. Biochemistry. 2000; 351: 769–777. PMC1221418 11042133

[46] D’Anna JA, Isenberg I Jr. Conformational changes of histone LAK (f2a2). Biochemistry. 1974; 13: 2093–2098. 4857059 10.1021/bi00707a015

[47] Ehrlich H, Hanke T, Frolov A, Langrock T, Hoffmann R, Fischer C, et al Modification of collagen in vitro with respect to formation of Nepsilon-carboxymethyllysine. Int J Biol Macromol. 2009; 44: 1: 51–6. 10.1016/j.ijbiomac.2008.10.001 18984004

[48] Barth A, Zscherp C. What vibrations tell us about proteins, Quarterly Reviews of Biophysics. 2002; 35: 4: 369–430. 12621861 10.1017/s0033583502003815

[49] Wells-Knecht KJ, Brinkmann E, Baynes JW. Mechanism of autoxidative glycosylation–identification of glyoxal and arabinose as intermediates in the autoxidative modification of proteins by glucose.J Org Chem. 1995; 60: 20: 6246–6247. 10.1021/bi00011a027 7893666

[50] Guedes S, Vitorino R, Domingues MR, Amado F, Domingues P. Glycation and oxidation of histones H2B and H1: in vitro study and characterization by mass spectrometry. Anal Bioanal Chem. 2011; 399: 10: 3529–39. 10.1007/s00216-011-4679-y 21274518

[51] Ishimaru D, Andrade LR, Teixeira LSP, Quesado PA, Maiolino LM, Lopez PM, et al Fibrillar aggregates of the tumor suppressor p53 core domain. Biochemistry. 2003; 42: 9022–9027. 12885235 10.1021/bi034218k

[52] Sattarahmady N, Moosavi-Movahedi AA, Habibi-Rezaei M, Ahmadian S, Saboury Heli N. Detergency effects of nanofibrillar amyloid formation on glycation of human serum albumin. Sheibani Carbohydr Res. 2008; 343: 2229–2234. 10.1016/j.carres.2008.04.036 18513709

[53] Ghosh S, Pandey NK, Singha Roy A, Tripathy DR, Dinda AK, Dasgupta S. Prolonged glycation of hen egg white lysozyme generates non amyloidal structures, PLoS One. 2013; 8:9: e74336 10.1371/journal.pone.0074336 24066139 PMC3774808

[54] Bouma B, Kroon-Batenburg LM, Wu YP, Brünjes B, Posthuma G, Kranenburg O, et al Glycation induces formation of amyloid cross-beta structure in albumin. J Biol Chem. 2003; 278: 43: 41810–9. 12909637 10.1074/jbc.M303925200

[55] Kuleva NV, Fedorova MA, Frolov AA. Dynamics of rat hepatocyte histone glyoxidation after general x-ray irradiation. Tsitologiia 2006; 48(6): 515–21. 16893058

[56] Tarkka T, Yli-Mäyry N, Mannermaa RM., Majamaa K, Oikarinen J. Specific non-enzymatic glycation of the rat histone H1 nucleotide binding site in vitro in the presence of AlF4-. A putative mechanism for impaired chromatin function. Biochim Biophys Acta 1993; 1180(3):294–8. 8422436 10.1016/0925-4439(93)90053-4

[57] Dixit K, Khan MA, Sharma YD, Moinuddin, Alam K. Peroxynitrite-induced modification of H2A histone presents epitopes which are strongly bound by human anti-DNA autoantibodies: role of peroxynitrite-modified-H2A in SLE induction and progression. Hum Immunol, 2011; 72(3): 219–25. 10.1016/j.humimm.2010.12.004 21182886

[58] Ansari NA, Dash D. Biochemical Studies on Methylglyoxal-Mediated Glycated Histones: Implications for Presence of Serum Antibodies against the Glycated Histones in Patients with Type 1 Diabetes Mellitus. ISRN Biochemistry Article ID 2013; 198065 10.1155/2013/198065 PMC4392999 25937957

[59] Khan MA, Dixit K, Moinuddin, Malik A, Alam K. Role of peroxynitrite-modified H2A histone in the induction and progression of rheumatoid arthritis. Scandinavian Journal of Rheumatology 2012; 41:426–33 10.3109/03009742.2012.698300 22985259

